# Structural properties of immune complexes formed by viral antigens and specific antibodies shape the inflammatory response of macrophages

**DOI:** 10.1186/s13578-024-01237-1

**Published:** 2024-04-25

**Authors:** Asta Lučiūnaitė, Kristina Mašalaitė, Ieva Plikusiene, Vincentas Maciulis, Silvija Juciute, Milda Norkienė, Aurelija Žvirblienė

**Affiliations:** 1 Institute of Biotechnology, Life Sciences Center, Vilnius University, Sauletekio Ave. 7, 10257 Vilnius, Lithuania; 2 NanoTechnas - Center of Nanotechnology and Materials Science, Faculty of Chemistry and Geosciences, Vilnius University, Vilnius, Lithuania; 3 State Research Institute Center for Physical Sciences and Technology, Vilnius, Lithuania; 4 Pharmacy and Pharmacology Center, Faculty of Medicine, Vilnius University, Vilnius, Lithuania

**Keywords:** Macrophage, Immune complex, Affinity, Inflammation

## Abstract

Data on the course of viral infections revealed severe inflammation as a consequence of antiviral immune response. Despite extensive research, there are insufficient data on the role of innate immune cells in promoting inflammation mediated by immune complexes (IC) of viral antigens and their specific antibodies. Recently, we demonstrated that antigens of human polyomaviruses (PyVs) induce an inflammatory response in macrophages. Here, we investigated macrophage activation by IC. We used primary murine macrophages as a cell model, virus-like particles (VLPs) of PyV capsid protein as antigens, and a collection of murine monoclonal antibodies (mAbs) of IgG1, IgG2a, IgG2b subclasses. The inflammatory response was investigated by analysing inflammatory chemokines and activation of NLRP3 inflammasome. We observed a diverse pattern of chemokine secretion in macrophages treated with different IC compared to VLPs alone. To link IC properties with cell activation status, we characterised the IC by advanced optical and acoustic techniques. Ellipsometry provided precise real-time kinetics of mAb-antigen interactions, while quartz crystal microbalance measurements showed changes in conformation and viscoelastic properties during IC formation. These results revealed differences in mAb-antigen interaction and mAb binding parameters of the investigated IC. We found that IC-mediated cell activation depends more on IC characteristics, including mAb affinity, than on mAb affinity for the activating Fc receptor. IC formed by the highest affinity mAb showed a significant enhancement of inflammasome activation. This may explain the hyperinflammation related to viral infection and vaccination. Our findings demonstrate that IC promote the viral antigen-induced inflammatory response depending on antibody properties.

**Supplementary Information:**

The online version contains supplementary material available at 10.1186/s13578-024-01237-1.

## Introduction

As demonstrated during COVID-19 pandemic, viral infection can cause severe inflammation [1]. Vaccination can also activate the immune system, leading to local or systemic adverse effects [2]. Antibodies (Abs) generated during viral infection or after vaccination opsonise viral antigens and form immune complexes (IC) [3]. These IC engage immune cells, such as monocytes/macrophages, by activating Fc receptors (FcRs) to remove the virus and activate effector functions, which determine the course of infection [4]. Normally, the cellular response induced by IC and FcR interaction tends to have an antiviral effect that mediates disease resolution. However, activation of innate immune cells can contribute to immunopathology due to severe inflammation, cell death, and complement activation [5]. The detailed mechanisms and key players in these processes are not clear. To avoid overactivation of the immune system, a comprehensive investigation of immune cell response to IC is essential. Therefore, our purpose was to investigate macrophage activation by IC composed of viral antigens and their specific monoclonal Abs (mAbs).

One of the immune cell activation mechanisms is via inflammasome formation. Inflammasomes are intracellular protein complexes representing important components of the innate immune system [6]. The best described representative is NLRP3 inflammasome. It contains three major components: nucleotide-binding and oligomerization domain-like receptor, apoptosis-associated speck-like protein containing a CARD (ASC) and pro-caspase-1. NLRP3 inflammasome assembly results in IL-1β release and inflammatory cell death [7]. Endogenous and external factors can trigger its assembly. NLRP3 inflammasome activation is associated with various inflammation-related diseases, including viral infection and Alzheimer’s disease [8]. NLRP3 inflammasome can be activated by disease-related oligomeric proteins, such as amyloid beta [9], α-synuclein [10], and tau [11] or various synthetic and natural particles, such as polymeric nanoparticles [12], cholesterol crystals [13], airborne pollutants [14]. Viral antigens can also mediate inflammasome activation, for example, viraporins [15] and structural proteins, as demonstrated with the nucleocapsid (N) protein of Zika virus [16] and severe acute respiratory syndrome coronavirus 2 (SARS-CoV-2) [17].

Recently, we investigated an inflammatory response induced by viral proteins of human polyomaviruses (PyVs) and paramyxoviruses in human macrophages [18]. We demonstrated that PyV-mediated inflammation was driven by NLRP3 inflammasome. Using viral proteins of different structures, we also showed that inflammatory responses, including inflammasome activation, depend on the structural properties of viral proteins. NLRP3 inflammasome can be activated by a variety of pathogen-associated factors or those related to cellular stress, therefore, it is one of the relevant mechanisms of innate immune cell activation by viral antigens. Moreover, IC promote phagocytosis of viral particles [19], and this may further result in inflammasome activation.

IC-induced cell signaling was shown to be different from that induced by the antigen alone, however, studies with IC were primarily based on classical IC models, such as ovalbumin or red blood cells [20, 21]. The role of IC of viral antigens in inflammasome activation is under investigation. It was shown that SARS-CoV-2 N protein-specific Abs produced in infected individuals can enhance inflammatory reactions related to inflammasome activation [19, 22]. Moreover, Abs can promote virus entry into monocytes via FcRs further enhancing inflammasome activation. These findings suggest that the IC formed by viral antigens and specific Abs may induce strong activation of innate immune cells.

Here, we aimed to investigate how viral antigens and their complexes with mAbs induce an inflammatory response in macrophages, focusing on inflammasome activation and secretion of inflammatory chemokines. We used recombinant VLPs derived from the major capsid protein VP1 of human Washington University (WU) PyV, self-assembling to spherical nanoparticles, and a set of VLP-specific mAbs. The cell response was compared between VLPs alone and IC to identify the pattern of inflammation induced by IC. IC properties and real-time formation were characterised using precise optical and acoustic surface-sensitive time-resolved techniques: spectroscopic ellipsometry (SE) and quartz crystal microbalance with dissipation (QCM-D). We demonstrated that the pattern of IC-induced cell activation was related to IC properties. These findings provide new insights into the IC-mediated inflammatory response.

## Materials and methods

### Materials

Dulbecco's modified Eagle's medium (DMEM; #31966047, United Kingdom), fetal bovine serum (FBS; #10500-064, United Kingdom), penicillin/streptomycin (P/S; #15140122, New York, USA), Dulbecco's Phosphate Buffered Saline (DPBS; #14190250, United Kingdom) were obtained from Gibco, Thermo Fischer Scientific. Cell culture plates: T75 culture flasks Cell Culture Treated EasYFlasks (#156499, Denmark) were from Nunc, Thermo Fischer Scientific; TPP Multi-well tissue culture plates (#92012, #92024, #92048, Switzerland) were from TPP Techno Plastic Products AG; IbiTreat 8-well μ-slides (#80826, Germany) were from Ibidi. MCC950 (#inh-mcc) was from InvivoGen (France). Horseradish peroxidase (HRP, #P8375, Germany) and dialysis tubes D-Tube Dialyzer Mini, MWCO 12–14 kDa, (#71505, Millipore, Ireland) were obtained from Sigma‒Aldrich by Merck. Hoechst 33342 (Hoechst, #639) from ImmunoChemistry Technologies (Minnesota USA). Dimethylsulfoxide (DMSO; #A3672) was from PanReac AppliChem and the ITW Reagents (Germany). NLRP3 silencing reagents: Opti-MEM Reduced Serum Medium (#31985070, New York, USA), Lipofectamine RNAiMAX Transfection Reagent (cat#13778030, California, USA), Silencer Select Negative Control No. 1 siRNA (cat#4390844, Lot ASO2KDRZ, 40 nmol, Texas, USA), Silencer Select Pre-designed siRNA NLRP3, assay ID s103710 (#4390816, Lot. ASO2KT00, 40 nmol, Texas, USA); chemiluminescent substrate SuperSignal West Pico PLUS (#34577, Illinois, USA) were from Thermo Fisher Scientific. Cell Lysis Buffer (#9803), Protease/Phosphatase Inhibitor Cocktail (100X, cat#5872) and anti-NLRP3 (D4D8T) Rabbit monoclonal antibody (#15101) were from Cell Signaling Technology (Massachusetts, USA). “NeA-Blue” 3,3′,5,5′-Tetramethylbenzidine (TMB) substrate (#01016-1-1000) was from Clinical Science Products (Massachusetts, USA). Bovine Serum Albumin (BSA, #P06-1391000) from PAN-Biotech (USA). RC syringe filter (cat#PA49.1), Tween-20 (#9127.1) and sulphuric acid (#X873.1) were from CarlRoth (Germany). Uncoated DuoSet ELISA kits for mouse CXCL2 (#DY452-05), CXCL1 (#DY453-05), CXCL9 (#DY492-05), CXCL16 (#DY503) and CCL8 (#DY790), Proteome Profiler Mouse Chemokine Array Kit (#ARY020) were from R&D systems (Minnesota, USA). Mouse IL-1 beta Uncoated ELISA Kit (#88-7013-77, Austria), TNF alpha Uncoated ELISA Kit (#88-7324-76, Austria), IL-10 Uncoated ELISA Kit (#88-7105-88, Austria), anti-β-Actin (BA3R) Loading Control Mouse mAb (#MA5-15739, Illinois, USA), CellMask Deep Red cell membrane stain (5 mg/ml stock, #C10046, Oregon, USA) and secondary antibodies: Donkey anti-Mouse IgG (H + L) Highly Cross-Adsorbed Secondary Antibody, Alexa Fluor 594, (#A21203, Oregon, USA); Goat anti-Mouse IgG (H + L) Highly Cross-Adsorbed Secondary Antibody, Alexa Fluor 488, (#A11029, Oregon, USA) were from Invitrogen, Thermo Fischer Scientific. Goat anti-Mouse IgG (H + L)-HRP conjugate (#1721011) and Goat Anti-Rabbit IgG (H + L)-HRP Conjugate (#1706515) were from Bio-Rad (California, USA).

### Cell culture

Primary macrophage cell culture was prepared from mouse brain macrophages (microglia) as described previously [9, 23]. Briefly, C57BL/6 mice used for primary microglia preparation were housed at Life Sciences Center of Vilnius University (Vilnius, Lithuania), which has State Food and Veterinary Service permissions to breed and use experimental animals for scientific purposes (veterinary certificate No. LT 59–13-001 and permission No. LT 61-13-004). The animals were grown in accordance with EU legislation of animal welfare law. Only the organs of animals were used, which did not require further approval. For microglia isolation, the brains of 0–3-day old mouse pups were used. Mature microglia were shaken off the astrocytic monolayer with a repetition of harvesting procedure every 2–3 days for up to three times. Cells were seeded at a density of 1 × 10^5^/cm^2^ in 1/2 old medium (conditioned medium from microglia shake) and 1/2 fresh DMEM supplemented with 10% FBS and 1% P/S and allowed to adhere overnight. On the next day, microglia were washed with serum-free DMEM and treated for 24 h in serum‐free DMEM containing 1% P/S. Cells were activated with mAbs at 7.5 µg/ml (50 nM) and WUPyV VLPs at 20 µg/ml (500 nM according to VP1 MW). Before cell treatment, each mAb and VLPs were mixed and incubated for 30 min at 37 °C to form IC. After 24 h of incubation, cell culture supernatants and cell lysates were collected, or the cells were assessed by immunocytochemistry. Cell culture supernatants and lysates were stored at − 20 °C for further cytokine or NLRP3 protein expression analysis.

Controls. Viral proteins were prepared in PBS, so, a control when PBS was added instead of viral proteins was used. MCC950, which selectively inhibits NLRP3 inflammasome, was used at 1 μM and added 30 min before the treatment.

### Production and characterisation of VLPs

Macrophages were treated with VLPs composed of up to 360 monomers of WUPyV recombinant major capsid protein VP1 (MW 40 kDa). VLPs were produced in *S. cerevisiae* yeast expression system and purified by CsCl density gradient centrifugation as described previously [24, 25]. The isolated VLPs were dissolved in PBS, dialysed, mixed with glycerol (1:1), filtered through a 0.2 µm pore size RC syringe filter and stored at -20 °C. VLP structure was verified by examination of the purified proteins using Morgagni-268 electron microscope (FEI, Inc., Hillsboro, OR, USA).

### Preparation of mAbs

For cell activation study and immunocytochemistry, in-house-generated murine mAbs against WUPyV VP1 VLPs were used (#mAb clone—subclass): #11D2—IgG1; #12F8—IgG2a; #4E12—IgG2a; #12F1—IgG2a; #5H10—IgG2b [25]. Hybridoma cells producing mAbs were cultured in DMEM supplemented with 15% FBS and 50 µg/ml gentamicin. The supernatant was collected at cell confluence and centrifuged at 1000 × g for 10 min to avoid cellular debris. Supernatants were assessed by ELISA for mAb specificity to WUPyV VP1 VLPs.

MAbs were purified from hybridoma cell culture supernatant by affinity chromatography using protein A (rProtein A SepharoseTM Fast Flow (GE Healthcare Bio-Sciences Corp., USA)). For IgG1 purification, the following buffers were used: elution buffer 100 mmol/L Gly at pH 3.0; washing buffers 1.5 mol/L Gly and 3 mol/L NaCl at pH 8.9. For IgG2a/IgG2b purification, the following buffers were used: elution buffer 100 mmol/L Gly, pH 3.0; washing buffer 1 mol/L Tris–HCl at pH 8.0, 0.1 mol/L Tris–HCl at pH 8.0, 0.01 mol/L Tris–HCl at pH 8.0. MAb purification was performed in several steps: column equilibration, sample application, column washing, and elution according to manufacturer’s instructions. Antibody fractions containing 0.1 mg/ml protein were collected. Buffer exchange to DPBS was performed in collected Ab fraction directly after purification. Abs were dialysed against DPBS using Amicon ultrafiltration system (100 kDa cutoff, cat#UFC5100BK, Sigma‒Aldrich by Merck) and filtered through 0.2 µm RC syringe filter. MAb concentrations were equalised to 3.7 mg/ml and stored at 4 °C. Concentration was evaluated with Nanodrop measuring absorbance at 280 nm and using IgG extinction coefficient ε = 1.37. The purity of mAbs was assessed by protein electrophoresis, and the specificity was determined by indirect enzyme-linked immunosorbent assay (ELISA) as described previously [25]. 5 μg/ml of VLPs was used for ELISA plate coating.

### Antibody labelling with horseradish peroxidase (HRP)

Purified mAbs were labelled with HRP by a periodate oxidation reaction as described previously [26]. MAb-HRP conjugates were dialysed against PBS using dialysis tubes D-Tube Dialyser and stabilised by adding 2% BSA and 50% glycerol and stored at – 20 °C. The activity of the conjugates was tested by direct ELISA. They were serially diluted, added to microtiter plates coated with VLPs and incubated for 1 h at RT. The plates were washed and developed with TMB as described in indirect ELISA. The dilution of mAb-HRP conjugates for competitive ELISA was selected from the titration curve corresponding to the half maximal effective concentration (EC50) determined by applying *Sigmoidal, 4PL, X is concentration* model and calculating EC50 with GraphPad Prism 10.1.0 software.

PBS—Phosphate Buffered Saline—0.0027 mol/L KCl, 0.0015 mol/L KH_2_PO_4_, 0.0081 mol/L Na_2_HPO_4_, 0.137 mol/L NaCl.

### Competitive ELISA

Microtiter plates (Nerbe Plus) were coated with 100 μl/well of VLPs diluted in the coating buffer (0.05 M sodium carbonate, pH 9.5) at a concentration of 1 μg/ml. Plates were incubated overnight at 4 °C. The coated plates were blocked with 250 μl/well of PBS containing 2% BSA for 1 h at room temperature (RT), then rinsed twice with washing solution (PBS with 0.05% Tween-20). The unconjugated mAbs were diluted to 5 µg/ml (excess concentration) in PBST (PBS with 0.1% Tween-20), added to the wells (100 μl/well) and incubated for 1 h at RT. Plates were washed 5 times and mAb-HRP in PBST was added to the wells (100 μl/well) for 1 h at RT. mAb-HRP dilution was selected according to EC50. Then, plates were rinsed 7 times with washing solution. The enzymatic reaction was visualised by the addition of 100 μL of “NeA-Blue” TMB substrate to each well. The reaction was stopped by adding 50 μL/well of 3.6% H_2_SO_4_ solution. OD was measured at 450 nm (reference wavelength 620 nm) with a microplate spectrophotometer. Competition between the mAbs was observed where the OD decreased compared to mAb-HRP control (only PBST was added in unconjugated mAb step). OD of mAb-HRP control was used as the maximum value (100%) to count competition effect in % of each antibody. OD of each antibody was divided by this maximum value and expressed as a percentage of mAb-HRP. 100% indicates no competition, 50% – moderate competition and 0% – full competition.

### Quantitation of cytokines and chemokines in cell culture supernatants

ELISA kits for the measurement of mouse cytokines—CXCL1, CXCL2, CXCL9, CXCL16, CCL8, IL-10, IL-1β and TNF-α—in cell culture supernatants were used. The kits are based on sandwich ELISA technique. Supernatants were diluted up to 1:200. All procedures were performed according to manufacturer’s protocols. In the last step, TMB substrate solution was added to each well. Plates were monitored for 15 min for colour development, the reaction in wells was stopped with 3.6% H_2_SO_4_ solution, and OD was measured at 450 nm with a reference wavelength of 570 nm using Multiskan GO microplate spectrophotometer. A standard curve was generated from cytokine standard and the cytokine concentrations were calculated in the samples.

To quantify the profile of secreted inflammatory chemokines, Proteome Profiler Mouse Chemokine Array Kit was used. All procedures were performed according to manufacturer’s protocols. Briefly, membranes precoated with capture Abs were blocked with Array Buffer 6 for 1 h at RT. One millilitre of each sample was mixed with 0.5 ml of Array Buffer 4 and 15 μL of reconstituted Detection Antibody Cocktail and incubated for 1 h at RT. The membranes were incubated with prepared sample/antibody mixtures overnight at 2–8 °C. After washing, membranes were incubated with streptavidin-HRP for 30 min at RT. After washing, HRP enzymatic reaction was developed using Chemi Reagent Mix incubating for 1 min at RT. Chemiluminescent signals were detected with ChemiDoc Imaging system (Bio-Rad, USA).

### Downregulation of NRPL3 gene using siRNA

To confirm NLRP3 activation by VLPs in primary microglia, NRP3 gene was downregulated using siRNA. siRNA targeting the NLRP3 gene was called NLRP3 siRNA and siRNA used as control was called negative siRNA. Cells were transfected with NLRP3 siRNA according to manufacturer’s recommendations. First, siRNA stock was prepared by diluting in nuclease-free water to obtain 100 µM siRNA stock solution, aliquoted and stored at –70 °C. The stock solutions did not undergo more than 10 freeze‒thaw cycles.

Transfection was carried out under antibiotic-free conditions. siRNA of 40 nM was used for transfection. One day before transfection, cells were plated in ½ DMEM supplemented with 1% P/S and 10% FBS, and ½ conditioned medium (1 × 10^6^ cells per well in 6-well plate). 6 h before transfection, cell culture medium was replaced with 2 ml DMEM (6-well plate) supplemented with 5% FBS without antibiotics. Cells were transfected using Lipofectamine RNAiMAX (further called Lipofectamine). For 2 wells to be transfected, siRNA-Lipofectamine (1:5) complexes were formed in the following way. First, Lipofectamine reagent was diluted 62.5 times in Opti-MEM medium. A 100 µM siRNA stock was diluted 625 times in Opti-MEM in separate tube and incubated for 5 min. Then, siRNA was combined with Lipofectamine (1:1 ratio, Lipofectamine added to siRNA). The complex was mixed gently and incubated for 20 min at room temperature. Negative control siRNA-Lipofectamine complexes were performed in the same way. 500 µL of siRNA-Lipofectamine complex was added to each well containing cells, reaching a final volume of 2.5 ml and a final RNA concentration of 40 nM. Cells were incubated for 24 h. The medium was replaced with 2 ml of DMEM supplemented with 5% FBS without antibiotics (6-well plate), and cells were incubated for another 24 h. After transfection, cells were washed once and treated with VLPs in serum-free DMEM without antibiotics (1 ml per well). Knockdown of NLRP3 expression was confirmed by Western blot (WB).

### SDS‒PAGE and western blot analysis

To determine NLRP3 protein expression, WB assay was applied. After treatment, cells were washed twice with ice-cold PBS and lysed with lysis buffer supplemented with protease inhibitors (150 µl/well in 6-well plate). Lysed cells were frozen and the next day the thawed samples were centrifuged at 20 000 × g for 10 min. Supernatant was collected and stored at -20 °C. The cell lysates were boiled in a reducing sample buffer and separated by 4–12% polyacrylamide gel (#NW04122BOX, Thermo Fisher Scientific) electrophoresis (PAGE) in MOPS SDS running buffer (#B0001 Thermo Fisher Scientific). Proteins from the SDS‒PAGE gel were blotted onto 0.45 µm nitrocellulose membrane (#LC2001, Thermo Fisher Scientific) by wet transfer. The membrane was blocked with 5% BSA in PBS for 1 h at RT and rinsed with TBST. The membrane was incubated with primary antibodies against human/mouse NLRP3 (1:1000) in TBST with 1% BSA overnight at 4 °C. Thereafter, the secondary antibodies Anti-Rabbit-HRP (1:3000) in TBST with 1% BSA were applied for 1 h at RT. The HRP enzymatic reaction was developed using SuperSignal West Pico PLUS chemiluminescent substrate. Then, the membrane was washed with TBST and incubated with loading control anti-β-actin (1:1000) in TBST with 1% BSA for 1 h at RT. Later, the secondary antibodies Anti-Mouse-HRP (1:5000) in TBST with 1% BSA were applied for 1 h at RT. HRP reaction was developed as mentioned above. Chemiluminescent detection was performed with Azure 280 imaging system. ImageJ program was used for quantitative analysis of WB.

### Immunocytochemistry for studying the uptake of VLPs by macrophages

To show IC uptake, VLPs and IC were stained separately. Cells were stained in IbidiTreat 8-well coverslips. After the treatment with VLPs and their IC, cells were washed with DPBS and fixed in 4% PFA dissolved in DPBS for 15 min and permeabilized with 0.1% Triton X**-**100 prepared in PBS for 10 min. Blocking solution—PBS containing 2% BSA was applied for 30 min followed by two washing steps. Secondary antibodies AlexaFluor 488 goat anti-mouse (1:1000) were added to the blocking solution and incubated for 2 h. After washing, the primary antibodies mouse anti-PyV VP1 VLPs (purified mAbs at 1 µg/ml) in DPBS solution were added and incubated overnight. The secondary antibodies AlexaFluor 594 donkey anti**-**mouse (1:1000) were applied for 2 h followed by two washing steps. Then, nuclear (Hoechst 33342 at 1 μg/ml) and Deep Red Cell Membrane (1:1000) stains were added for 30 min. After washing, the cells were stored in DPBS. 3D images of 184.52 × 184.52 µm were taken using 63x/1.40 OIL HC PL APO CS2 objective with confocal Leica SP8 fluorescence microscope and TCS SP8 software (Leica Microsystems, Germany). Hoechst33342 signal was taken with excitation/emission wavelengths—405/407–480 nm (HyD detector), AlexaFluor 488 signal—492/498–578 nm (PMT detector), AlexaFluor 594 signal—594/599–646 nm (HyD detector), Deep Red Cell Membrane stain signal—641/650–784 nm (PMT detector), using sequential scan between lines. Acquired images were processed using ImageJ (Wayne Rusband; National Institute of Health, Bethesda, MD, USA). 3D reconstructions with orthogonal views were presented from 28 frames (see Additional file 1: Fig. S1 for more details). Z-stack images had z-size 8.06 μm and 28 frames with z-step size 0.3 μm (system optimised).

### VLP-mAb interaction study

The affinity interaction of VLPs with their specific mAbs was assessed using two combined surface-sensitive methods: SE and QCM-D. VLPs were covalently immobilised on the surface of the gold-coated QCM-D sensor disc pre-modified with the self-assembled monolayer consisting of 11-mercaptoundecanoic acid and 6-mercapto-1-hexanol. VLPs at a concentration of 500 nM in DPBS were injected into the measurement chamber for 60 min for covalent immobilisation. Then, measurement chamber was washed with PBS for 10 min. Subsequently, 100 nM mAbs (clones 11D2, 12F8, 4E12 and 5H10) in DPBS were used for the time-resolved interaction study. The IC of VLPs and each mAb were formed for 60 min.

IC formation via the interaction between immobilised VLPs and their specific mAbs was time-resolved simultaneously measured by changes in frequency (protein and buffer mass, QCM-D), energy dissipation (viscoelastic properties, QCM-D), and optical signal (SE). SE measurement provided information about surface saturation by proteins measuring the refractive index (n) of the formed protein layers and was used to calculate thermodynamic parameters.

### Statistical analysis

Statistical analysis was performed with GraphPad Prism 10.1.0 software (GraphPad Software, Inc., La Jolla, CA). The data are presented as box plots (showing minimum, first quartile, median, third quartile and maximum) or bar graphs (mean and SD) with individual data points of at least 4 independent experiments (N) indicating the number of independent cell culture preparations. Normality test was carried out to test whether the values came from a Gaussian distribution. Statistical comparisons of treatments were performed with one‐way ANOVA in conjunction with Tukey’s multiple comparison test or Student’s t test. A Kruskal–Wallis test with Dunn's post hoc test was used for nonparametric data. Differences with p values less than 0.05 were considered to be statistically significant: *p < 0.05, **p < 0.01, ***p < 0.001, ****p < 0.0001. To emphasise nonsignificant results ns was used.

## Results

### Characterisation of mAbs and IC formation

#### Analysis of VLP-specific mAb epitopes by competitive ELISA

**Fig. 1:**
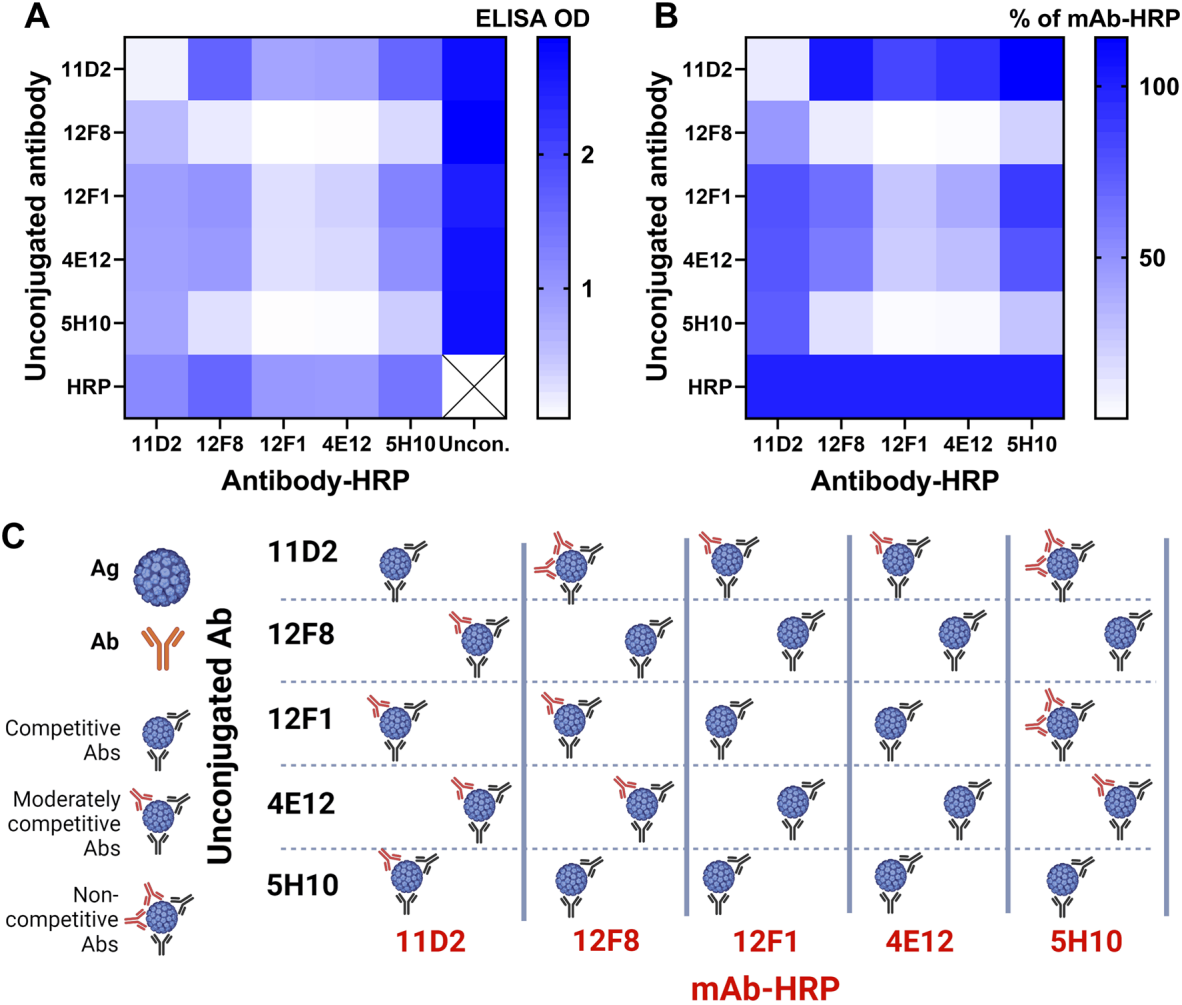
Characterization of mAbs according to their epitopes. Competition of each mAb to bind the same epitope was evaluated by competitive ELISA. Firstly, unconjugated mAbs were added followed by mAb-HRP addition. OD values represent binding efficacy of mAb-HRP in the presence of unconjugated mAbs. OD of each mAb-HRP control was set as maximum value (100%) to evaluate the competition effect of unconjugated mAbs. The OD of each mAb (represented in **A**) was divided by this maximum value and expressed as the percentage of mAb-HRP binding (% of mAb-HRP in **B**). 100% indicates no competition, 50% – moderate competition and 0% – full competition/no binding. (**C**) Schematic representation of mAb (mAb-HRP) binding to VLPs in the presence of another mAb (unconjugated mAb). The absence of mAb-HRP (red) indicates that the epitope is fully occupied by another mAb (black, unconjugated mAb), one mAb-HRP indicates that mAb-HRP binds to adjacent epitope of unconjugated mAb epitope, and two mAb-HRP indicates that mAb-HRP binds to epitopes different from those of the unconjugated mAbs. **C** was created with BioRender.com, AG No.: ES268ORVA0

To compare the binding sites of mAbs obtained from different hybridoma clones, a competitive ELISA was performed (Fig. 1). All mAbs bind the quaternary structure of WUPyV VLPs and all mAbs, except 11D2 (IgG1), recognise denatured VP1 of WUPyV [25]. MAb 11D2 binds only the quaternary structure of WUPyV VLPs. Competitive ELISA also confirmed that 11D2 recognises a conformational epitope (Fig. 1). When mAbs 11D2 are bound to VLPs, the other mAbs can still bind their epitopes, as they occupy distinct VLP places. Interestingly, once the other mAbs bind to VLPs, lower amounts of 11D2 can associate VLPs, although the presence of 11D2 interferes only with 12F1 (IgG2a) and 4E12 (IgG2a) but not with 12F8 (IgG2a) and 5H10 (IgG2b) (Fig. 1C). mAbs 12F8 and 5H10 recognise similar epitopes, as they compete for VLP binding (Fig. 1C). In addition, the other mAbs, except mAb 11D2, are also unable to associate VLPs after binding of either 12F8 or 5H10 mAbs (Fig. 1C). However, both mAbs, 12F8 and 5H10, can bind to VLPs if either 4E12 or 12F1 mAbs are bound to VLPs. This shows that mAbs 12F8 and 5H10 recognise different epitopes than 4E12 and 12F1. It could be that 12F8 and 5H10 induce conformational changes in VLPs or occupy many VLP epitopes, preventing the binding of mAbs 4E12 and 12F1. Meanwhile, mAbs 12F1 and 4E12 fully compete with each other, which suggests the same epitope. Therefore, only 4E12 was selected for further characterisation. Summarising the results of competitive ELISA, mAbs 4E12 and 12F1 recognise the same or overlapped epitopes, 12F8 and 5H10 – closely located epitopes, 11D2 – a completely different epitope, and a group of mAbs 4E12, 12F1, 12F8 and 5H10 – the same or closely located epitopes.

#### Real-time analysis of IC formation and antibody affinity by SE and QCM-D

**Fig. 2:**
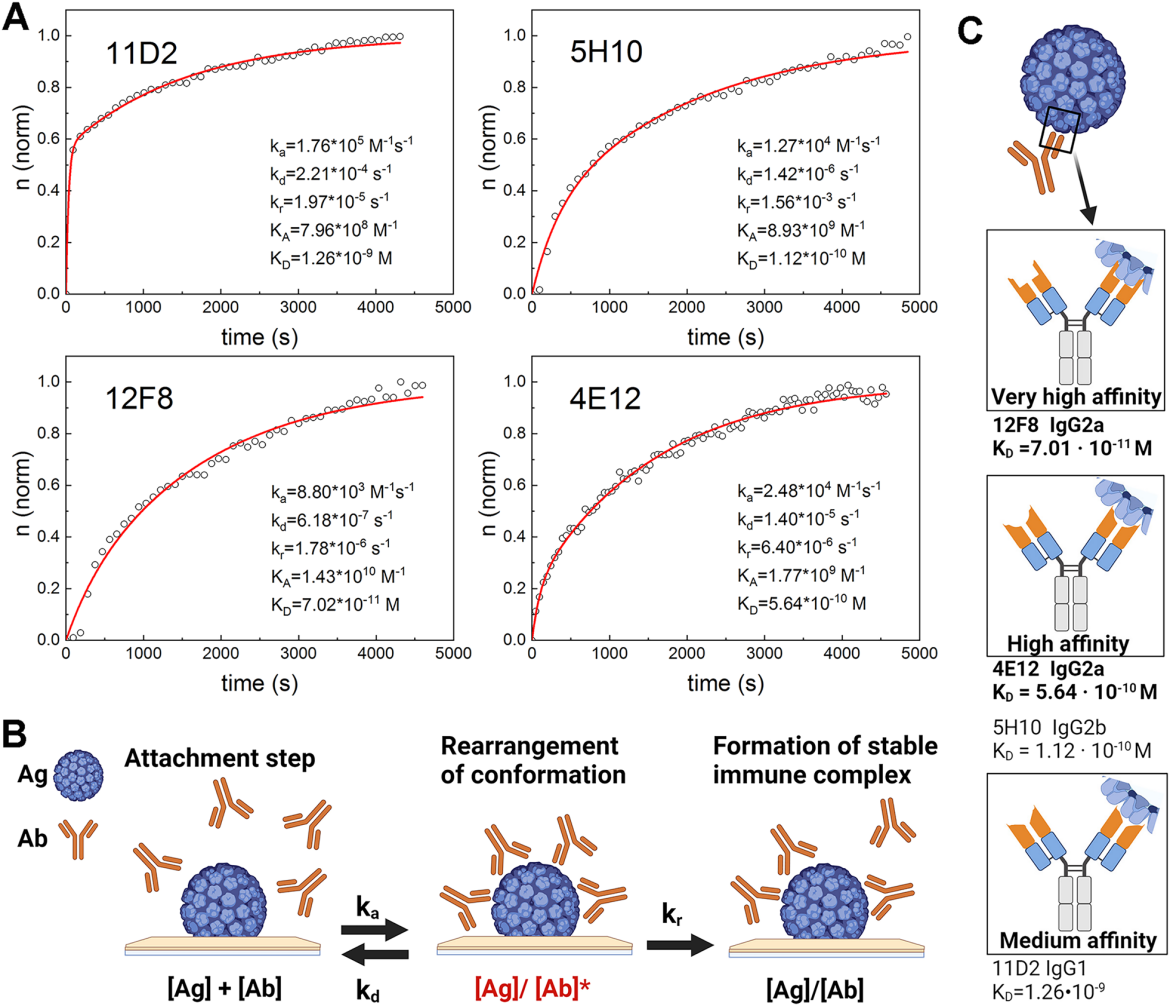
The affinity of mAbs determined by SE. **A** Real-time interaction of mAbs with covalently immobilised VLPs, n – refractive index of the formed layer. n values as time functions are represented in the graphs. 11D2, 12F8, 4E12 and 5H10 refer to mAb clones. **B** Schematic representation of IC formation steps, VLPs referred as Ag antigen. **C** Schematic representation of VLP (antigen) binding to mAbs of various affinities. The affinities of mAbs were expressed in K_D_ and are represented for each mAb. K_D_ values of IgG2a mAbs are indicated in bold. Kinetic parameters—rate constants: k_a_ association, k_d_ dissociation, k_r_ stable IC formation; K_A_ equilibrium association constant, and K_D_dissociation constant. **B** and **C** were created with BioRender.com, AG No.: OZ268OSOXN and AG No.: XD268OT4QA

To determine mAb affinity and thermodynamic parameters, high-sensitivity biosensing techniques – SE and QCM-D—were applied. SE measures the change in polarised light state upon reflection from the sample. It allows to observe changes in refractive index (n) and thickness (d) of the protein monolayer on the sensing surface during their formation. The acoustic method QCM-D measures changes in frequency (ΔF) and energy dissipation (ΔD) and provides information about protein monolayer viscoelastic properties simultaneously with SE. The equilibrium dissociation affinity constants (K_D_) and binding rate constants (k_a_) were obtained from the measurement of interaction kinetics using SE and fitting normalised *n* evolution in time with consequent binding mathematical model based on encounter theory (Fig. 2A, Table 1) [30–33]. mAbs were divided into three groups according to their affinity (K_D_ value): medium, high and very high (Fig. 2C). 12F8 was distinguished with very high affinity, 5H10 and 4E12 were distinguished with high affinity and 11D2—medium affinity. Thus, mAb affinity characterisation showed their differences and supplemented competitive ELISA data. The slightly different affinities of mAbs 12F8 and 5H10 confirm the differences of these mAbs, although they bind closely located VP1 sites according to competitive ELISA data.

**Table 1:** Thermodynamic properties and surface mass density of IC formation obtained by SE using VLPs and mAbs of different clones

Γ^SE^, ng/cm^2^	k_a_, M^–1^ s^–1^	k_d_, s^–1^	k_r_, s^–1^	K_A_, M^–1^	K_D_, M
WUPyV VLP					
392	–	–	–	–	–
IgG1 mAb (clone 11D2)					
51.3	1.76 · 10^4^	2.21 · 10^–4^	1.97 · 10^–5^	7.96 · 10^8^	1.26 · 10^–9^
IgG2a mAb (clone 12F8)					
44.9	8.80 · 10^3^	6.18 · 10^–7^	1.78 · 10^–7^	1.43 · 10^10^	7.01 · 10^–11^
IgG2a mAb (clone 4E12)					
28.4	2.48 · 10^4^	1.40 · 10^–5^	6.40 · 10^–6^	1.77 · 10^9^	5.64 · 10^–10^
IgG2b mAb (clone 5H10)					
60.9	1.27 · 10^4^	1.42 · 10^–6^	1.56 · 10^–6^	8.93 · 10^9^	1.12 · 10^–10^

Γ^SE^ dry protein surface mass density, k_a_ association rate constant, k_d_ dissociation rate constant, k_r_ the stable immune complex formation rate constant, K_A_ the equilibrium association constant, and K_D_ dissociation constant

The association rate constant (k_a_) data distinguished 12F8, as the highest amount of this mAb bound to VLPs per second compared to other mAbs (Table 1). The k_a_ values of the other mAbs were of the same grade (10^4^), although mAb 5H10 associated with VLPs faster than the others. The dissociation rate constant (k_d_) also showed that 12F8 dissociates faster than the other mAbs (Table 1). We also calculated the stable IC formation rate constant (k_r_), which corresponds to the time needed to form a tightly bound IC (Table 1). k_r_ value indicated that 12F8 needed the longest time to form a stable IC. These data show that very high-affinity mAbs bind antigens particularly fast, however, the formation of stable IC is a time-consuming process.

The dry protein surface mass density (Γ^SE^) demonstrates the quantity of mAbs bound to specific antigen and was calculated from SE measurements under steady-state conditions (Table 1). The lowest Γ^SE^ = 28.4 ng/cm^2^ was calculated after IC formation by mAb 4E12 and VLPs. The IC formed by mAbs 11D2 and 5H10 distinguished themselves with the highest surface mass density value among all investigated mAbs (Γ^SE^ = 51.3 ng/cm^2^ for 11D2 and Γ^SE^ = 60.9 ng/cm^2^ for 5H10). The highest affinity mAb 12F8 was in the middle according to mAb mass on VLP layer. (Γ^SE^ = 44.9 ng/cm^2^).

**Fig. 3:**
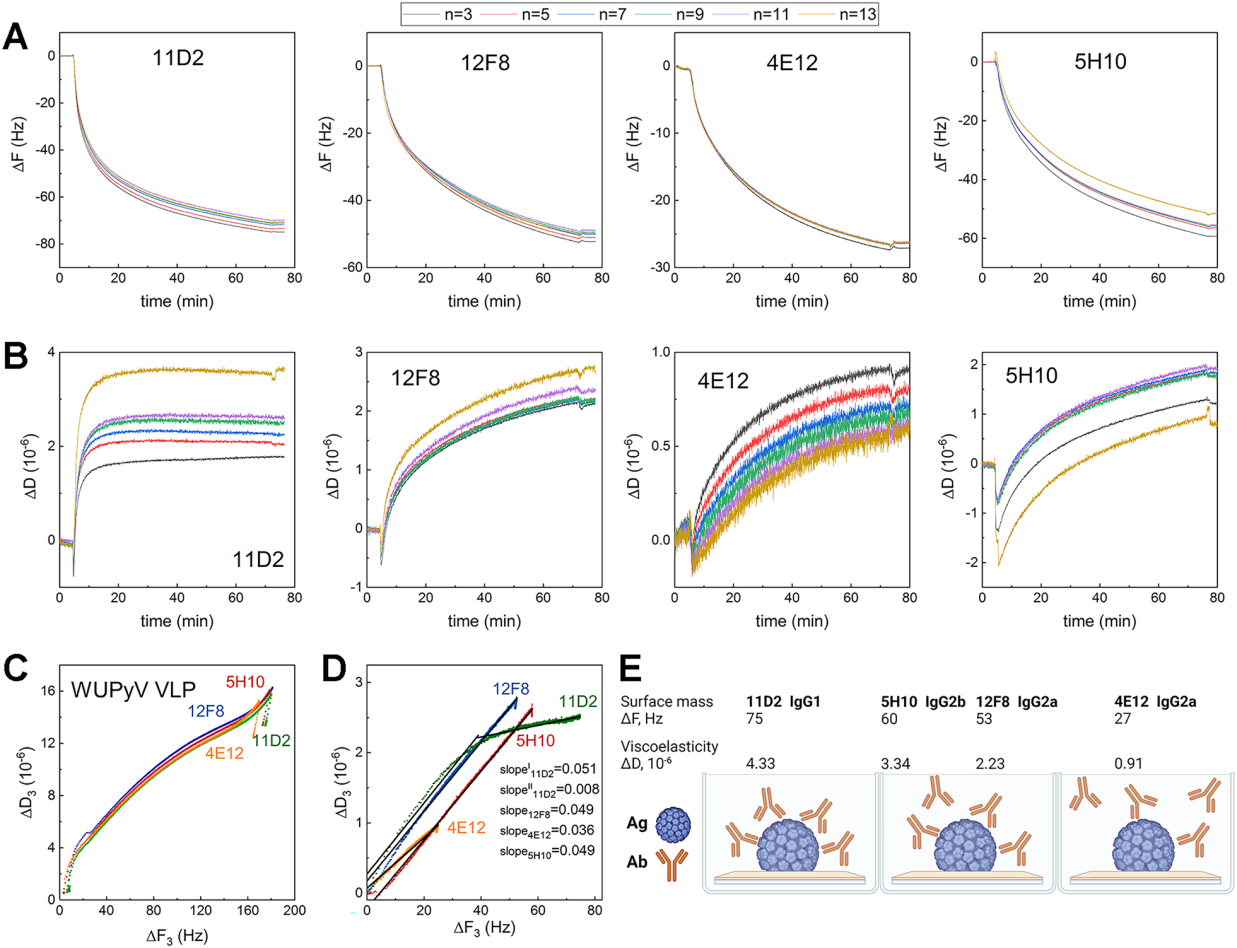
Characterisation of IC formation by QCM-D. Time-resolved changes in (**A**) ΔF (frequency change proportional to surface mass) and (**B**) ΔD (dissipation change proportional to viscoelastic properties) during IC formation with mAbs 11D2, 12F8, 4E12 and 5H10. n—number of measured overtones of oscillating quartz crystal. ΔD/ΔF plots obtained by plotting ΔF on the x-axis and ΔD on the y-axis and eliminating time as an explicit parameter for antigen (Ag) VLPs (**C**) and for mAbs of different clones 11D2, 12F8, 4E12 and 5H10 (**D**). (C) VLPs were covalently immobilized on the gold surface for each mAb (clones 11D2, 12F8, 4E12 and 5H10) and ΔD/ΔF plots for separate VLP immobilization are shown. **D** The slopes were calculated from fitted lines of ΔD/ΔF plots for mAbs. If ΔD/ΔF plot is non-linear (more than 1 slope) it indicates conformational changes on the layer during VLP-mAb interaction. From fitted lines the slopes were calculated (**D**). **E** Schematic representation of formed IC layer characteristics. ΔF values represent the changes in surface mass of mAbs bound to VLPs (Ag). **E** was created with BioRender.com, AG No.: AV268OTFM3

Simultaneously with SE, we used QCM-D to investigate the viscoelastic properties (ΔD) of the formed mAb layer that can be related to conformational changes after IC formation. The time-resolved ΔF and ΔD changes during IC formation by mAbs and VLPs are presented in Fig. 2. ΔF is proportional to the change in the surface mass density of the layer containing mAbs and entrapped buffer molecules, while SE provided information on the dry protein mass density. According to the changes in ΔF and ΔD, different amounts of mAbs (ΔF) and viscoelastic properties (ΔD) were observed for the IC of different mAbs (Fig. 3D, E). The lowest change in ΔF was observed for mAb 4E12 after binding to VLPs. The highest amount of bound mAbs was detected in the IC formed by mAbs 11D2 and 5H10 (Fig. 3A). This suggests that these IC have the highest coverage by mAbs, which is in agreement with SE data of dry protein mass (Γ^SE^, Table 1). The ΔD obtained for mAb 4E12 during its interaction with VLPs reached 1*10^–6^ after 60 min, demonstrating that this mAb layer has low viscoelastic properties and forms a rigid monolayer compared to other mAbs. These findings also show that the formation of IC with mAb 4E12 is a relatively static process. The slope value calculated for the formation of IC with 4E12 was slope_4E12_ = 0.036, which is similar to other rigid layer formation slope values [34]. Contrarily, IC formation with another IgG2a subclass mAb 12F8 was a more dynamic process, as the determined ΔD for this monolayer was 2.5*10^–6^ after 60 min with possible conformational changes. This also explains competitive ELISA data – once mAb 12F8 is bound to VLPs, it is more difficult to attach other mAbs to VLPs, even if their epitopes are completely different, as in the case of mAb 11D2.

The ΔD demonstrated that IC formation by mAb 11D2 was the most dynamic process, characterised by relatively high viscoelastic properties (ΔD = 3.5*10^–6^) of the formed mAb layer after steady-state conditions were reached (Fig. 3B). The high change in ΔD during mAb-VLP interaction is also due to the higher increase in the surface mass density. The slope values were slope_12F8_ = 0.049, indicating a high ΔD value per ΔF unit and viscoelasticity of this IC. The IC formed by mAb 5H10 was characterised by higher ΔD in comparison to the IC formed by mAb 12F8. According to IC formation curve (Fig. 3D), 5H10 and 12F8 had the same curve profile, and bound mAb mass was directly proportional to dissipation, suggesting a constant IC formation process. The calculated slope value for mAb 5H10 was slope_5H10_ = 0.049, the same as for IC formed by 12F8. Thus, mAbs 12F8 and 5H10 had very similar dynamics of IC formation what is expected for mAbs with similar epitopes. In contrast, IC with 11D2 was characterised by uneven formation (Fig. 3D), suggesting that this mAb had conformational changes and the highest dynamics during IC formation process. Real-time monitoring of ΔD showed two steps of this IC formation characterised by two slopes in the dissipation curve (Fig. 3D). Since the initial values are comparable with other mAbs at slope^I^_11D2_ = 0.051, the formation of this IC had similar viscoelastic properties as the other IC. The second slope value slope^II^_11D2_ = 0.008 indicates a substantial reduction (approximately 6 times) in ΔD values per ΔF, showing conformational changes of already bound mAb 11D2 molecules. At the beginning, this IC formation was very fast (exhibited by spread data points in the 0–25 Hz interval), however, this process slowed down as there was less free surface for IC formation (exhibited by more densely place data points from 25 to 75 Hz), indicating conformational changes.

### Macrophage response to VLPs and IC

#### VLPs and IC induced release of inflammatory cytokines and chemokines in macrophages

To investigate macrophage activation by IC composed of mAbs and viral antigens, we used a collection of murine mAbs generated against WUPyV-derived VLPs by hybridoma technology [25]. Previously, we demonstrated that recombinant VLPs of human PyVs induce inflammatory response in human and murine monocyte-derived macrophages [18, 27]. We also showed that the IC formed by mAbs and WUPyV VLPs mediate a similar response as VLPs alone [27]. Furthermore, we revealed inflammasome activation by amyloid beta oligomers using murine microglia cells [9]. Based on these findings, we selected murine microglia as a macrophage model to explore inflammasome activation by IC. To prove the relevance of the selected cell model, we compared the secretion of inflammatory cytokines by VLP-treated microglia to previously determined cytokine secretion profiles in monocyte-derived macrophages [18, 27]. We detected that WUPyV VLPs induced TNF-α (Fig. 5A, Additional file 1: Fig. S2A), IL-12/23 (Additional file 1: Fig. S2C), and IL-1β release (Fig. 5B and Additional file 1: Fig. S2B) in microglia cells as previously detected in in monocyte-derived macrophages. Thus, murine microglia culture represents a suitable model for studying macrophage activation by IC composed of mAbs and VLPs.

**Fig. 4:**
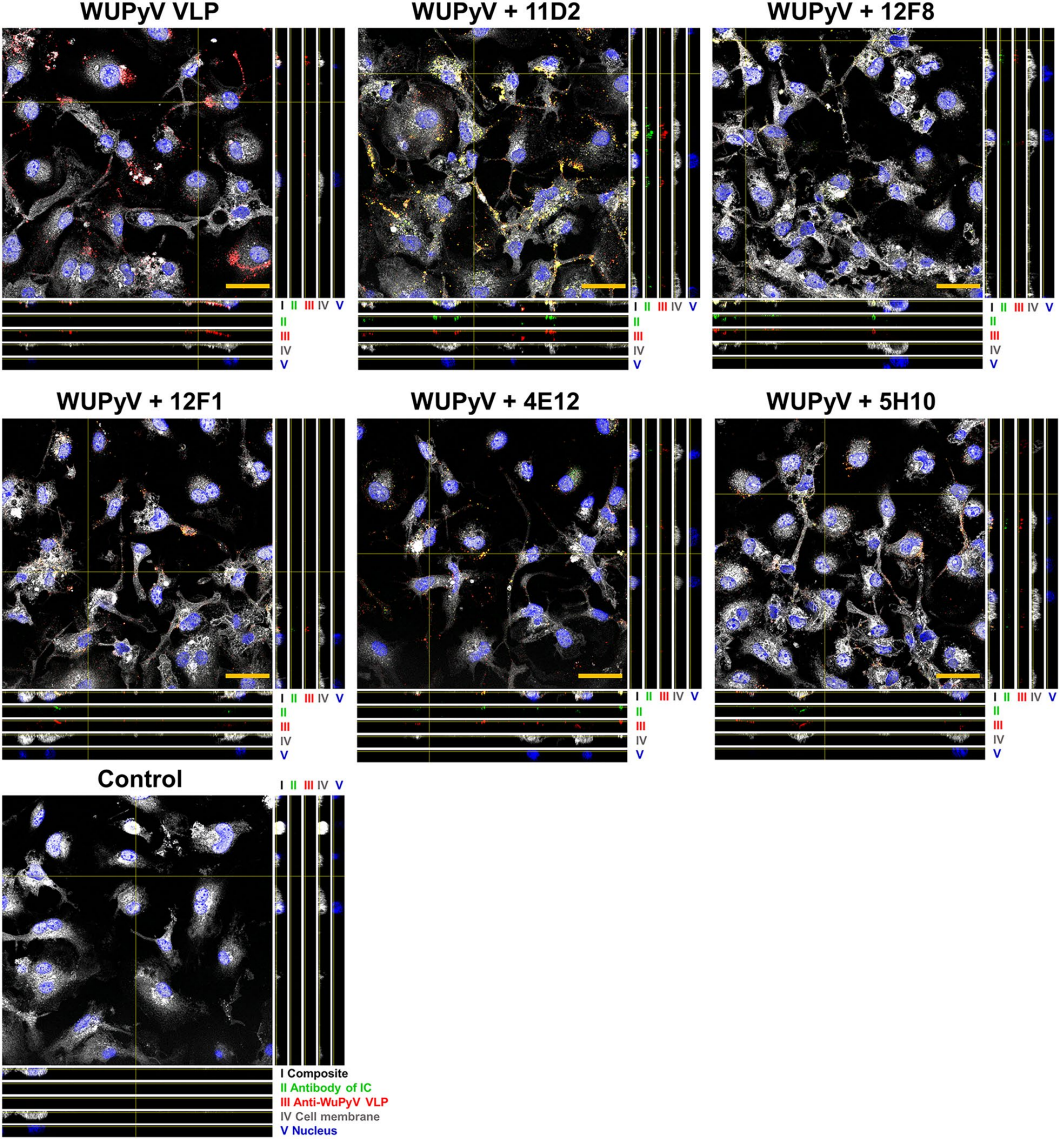
IC formation and uptake by macrophages. Cells were treated with VLPs (20 µg/ml) and mAbs (7.5 µg/ml) for 24 h and stained with VLP-specific MAbs, cell stain (gray) and nuclear stain Hoechst33342 (blue). To detect the IC, VLPs and IC were stained separately. Firstly, IC were stained with secondary Ab-AlexaFluor 488 (green; antibody of IC), then anti-WuPyV VP1 followed by secondary Ab-AlexaFluor 594 (red). The experiment was repeated 3 times, and representative images of one experiment are shown. Composite images of z = 4 (frame) of 28 frames and orthogonal views are shown, separate channel images are shown in Fig. S1D, E. Z-projects of composite and separate channel images are represented in Fig. S1 A-C. 3D images of 184.52 × 184.52 µm and z-size 8.06 μm were taken using 63 × oil objective with Leica TCS SP8 confocal microscope. The scale bars—30 μm. 11D2, 12F8, 4E12, 5H10—mAb clones

To investigate IC-induced cell activation, we focused on inflammasome activation and viral infection-related chemokines. First, we characterised IC uptake by microglia cells (further called macrophages). The VLPs and IC-forming mAbs were fluorescently stained separately in the cells and their signal was detected by a confocal microscope. Co-localisation of VLP and mAb signals showed that the formed IC were taken up by macrophages (Fig. 4 and Additional file 1: Fig. S1), demonstrating their interaction with the cells.

**Fig. 5:**
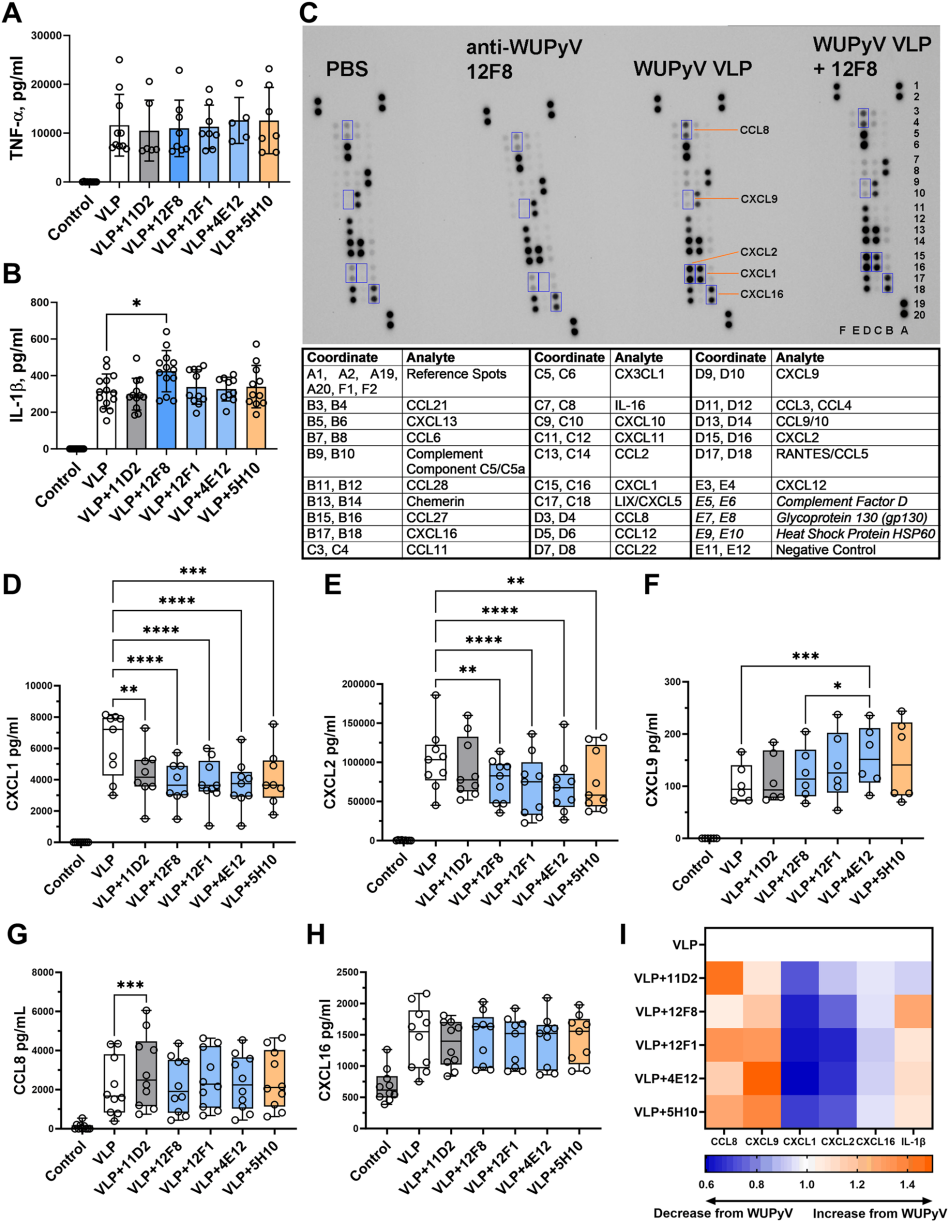
VLPs and IC induced different release of inflammatory molecules in macrophages. Cells were treated with VLPs (20 µg/ml) and mAbs (7.5 µg/ml) for 24 h. **A** TNF-α (N = 5–10) and **B** IL-1β (N = 10–14) secretion determined by ELISA. **C** Chemokine secretion assessed by Proteomic Chemokine Profile Array Kit. Four membranes combined into one image are shown. They represent control (PBS), antibody control (mAb 12F8 alone), VLP control (WUPyV VLPs) and IC formed by WUPyV VLPs and mAb 12F8 treatments. Blue rectangles represent selected chemokines for determination by ELISA. Chemokine coordinates are presented in the table below the blot, *in italics* are sample controls—proteins commonly present in cell culture supernatants and used as positive signals. **D** CXCL1 (N = 8–9), **E** CXCL2 (N = 9), (**F**) CXCL9 (N = 6), (**G**) CCL8 (N = 10), (**H**) CXCL16 (N = 9) secretion determined by ELISA. (**I**) Representation of IC-induced chemokine secretion levels compared to VLPs. Average concentrations of detected chemokines were normalised to VLP-induced values to evaluate the changes from VLP effect, which is equal 1.0. The arrows show higher (orange) or lower (blue) chemokine secretion compared to VLPs. Data are represented using bar graphs or box plots with dots showing the number of independent experiments (N), *p < 0.05, **p < 0.01, ***p < 0.001, ****p < 0.0001, one-way ANOVA followed by Tukey’s multiple comparison test, for chemokines test option of matched measures across one N was used. 11D2, 12F8, 4E12, 5H10—mAb clones

Furthermore, the inflammatory response induced by VLPs and their IC was evaluated by measuring TNF-α release, indicating activation of NF-κB pathway, and IL-1β release, indicating inflammasome activation (Fig. 5A, B and Additional file 1: Fig.S2A, B). We found that VLPs induced the release of investigated cytokines. No differences were observed in TNF-α secretion levels compared to macrophages treated either with VLPs alone or IC (Fig. 5A). In contrast, we found that IC promoted a higher release of IL-1β than VLPs alone, although only IC formed by 12F8 (IgG2a subclass) induced a significantly higher level of IL-1β (Fig. 5B). We also measured the level of the anti-inflammatory cytokine IL-10 after VLP and IC treatments, however, we did not detect any IL-10 secretion (the signal was below the assay detection limit).

To investigate whether IC induced other inflammatory processes, we evaluated the secretion of inflammatory chemokines. First, we used Proteome Profiler Mouse Chemokine Array Kit to determine secreted chemokines after treatment with VLPs and the IC formed by mAb 12F8 (Fig. 5C). We observed a release of several chemokines at the basal level (control) and a higher release after VLP and IC treatments. Based on these data, we selected several chemokines – CXCL1, CXCL2, CXCL9, CXCL16, CCL8 – for further evaluation by ELISA. Our selection was also based on previous data on chemokine secretion by mouse macrophages/microglia during viral infection [28] and their classification to inflammatory chemokines in both mice and humans [29]. We found a significant induction of selected chemokines by VLPs (Fig. 5D–I) and Additional file 1: Fig. S2D–H). IC formed by different mAbs either downregulated or upregulated the secretion of selected inflammatory chemokines. All IC downregulated the release of chemokines CXCL1 and CXCL2, which regulate inflammasome activation (Fig. 5D, E, I). However, the decrease in CXCL2 secretion after cell treatment with IC of IgG1 mAb was insignificant (Fig. 5E, I). Secretion of CXCL9, implicated in strong antiviral response, was upregulated by IC treatment compared to VLPs alone, although the highest and significant increase in CXCL9 release was detected only in cells treated with IC of IgG2a mAb 4E12 (Fig. 5F, I). The secretion of another inflammatory chemokine CCL8 was significantly upregulated only by IC of IgG1 mAb 11D2 (Fig. 5G, I). VLPs also promoted the secretion of lymphocyte attracting chemokine CXCL16, however, we did not observe any differences between IC- and VLP-induced CXCL16 levels (Fig. 5H). This chemokine, supposed to be secreted in inflammatory conditions, was also released at basal level in cell culture (Fig. 5H).

#### The impact of IC properties on the IC-mediated inflammatory response

Further, we compared mAb affinity and the IC-induced secretion of inflammatory molecules in macrophages. We found that only IC formed by the highest affinity mAb (12F8) significantly enhanced IL-1β release (Fig. 5B). The investigated IC also mediated the release of inflammatory chemokines depending on IC features. Despite the different affinities of mAbs, all IC downregulated the release of CXCL1 and CXCL2, except the IC formed by IgG1 mAb (11D2), which did not change CXCL2 secretion (Fig. 5I). Furthermore, only IC formed by mAb 11D2 significantly upregulated CCL8 release (Fig. 5G), evincing this IC uniqueness. CXCL9 release was significantly upregulated by IC of mAb 4E12 and it was significantly different compared to IC of higher affinity mAb 12F8 of the same subclass IgG2a (Fig. 5F). Another difference between mAbs 4E12 and 12F8 was their coverage of VLPs (ΔF, Fig. 3E). More mAb 12F8 was bound to VLPs during IC formation, which could also influence cell activation profile. Comparing IC formed by IgG2a/b mAbs, we observed a tendency that the lower affinity of mAb, the higher CXCL9 release was induced compared to VLP-mediated effect (Fig. 5I, 2C). Our results show that the subclass and especially the affinity of mAbs have a significant impact on the IC-induced inflammatory response.

### Relation of inflammasome activation and IC characteristics

**Fig. 6:**
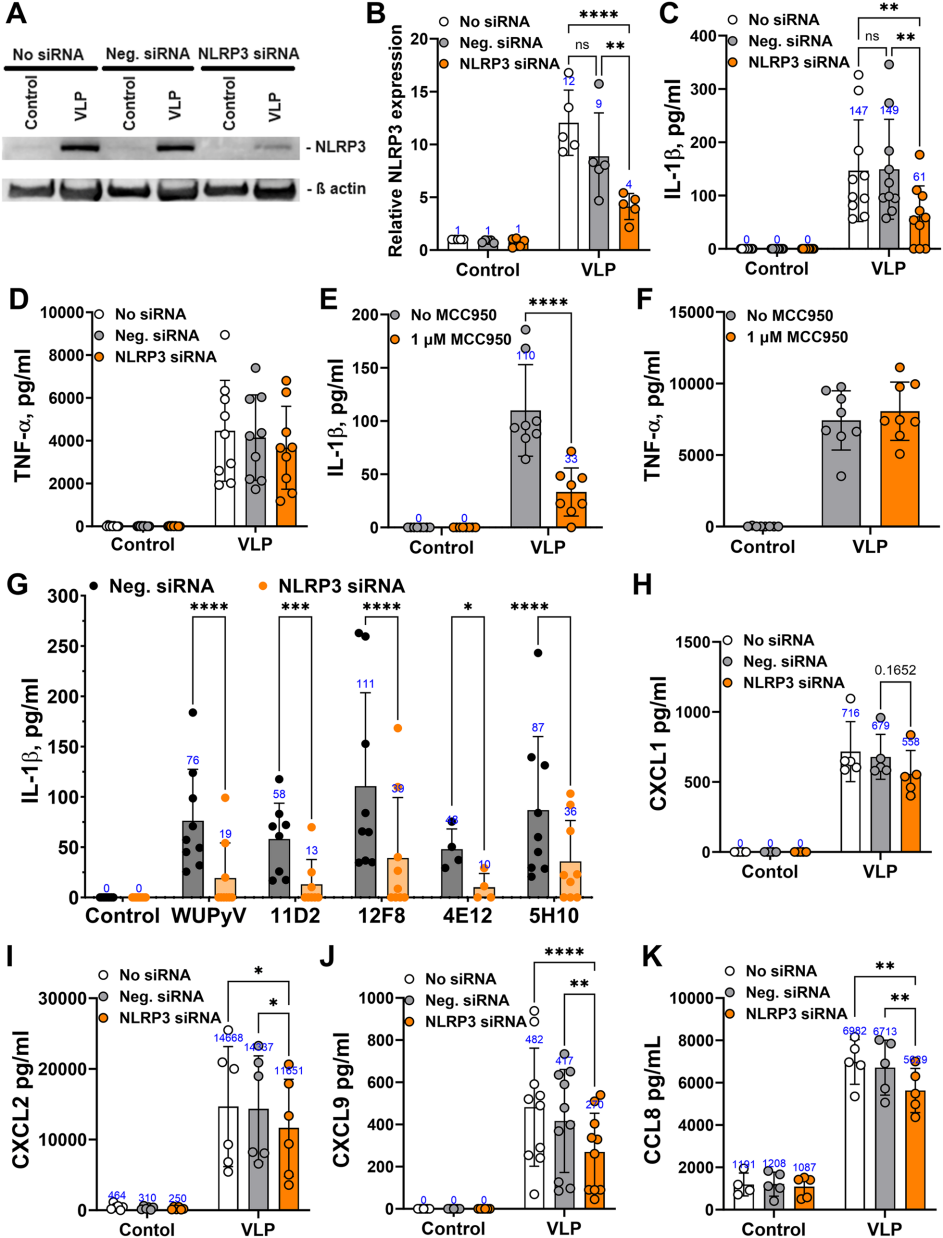
VLP-induced release of inflammatory molecules is related to NLRP3 inflammasome activation. Macrophages were treated with VLPs (20 µg/ml) and mAbs (7.5 µg/ml) for 24 h. siRNA was used to downregulate *NLRP3* gene expression, negative (neg.) siRNA was used as a control. WB of NLRP3 protein expression: **A** representative image, **B** quantification (N = 5), to calculate relative NLRP3 expression NLRP3 protein signal was normalized to β-actin signal and the calculated value was divided by control value in the absence of siRNA (No siRNR equal to 1.0 of every independent experiment). **C**, **E** IL-1β (N = 10 and 8) and **D**, **F** TNF-α (N = 9 and 8), (**H**) CXCL1 (N = 5), (**I**) CXCL2 (N = 6), (**J**) CXCL9 (N = 10), (**K**) CCL8 (N = 5) secretion determined by ELISA. Inhibitor MCC950 to block NLRP3 inflammasome activation was used at a concentration of 1 μM and added 30 min before the treatment (**E**). (**G**) IL-1β (N = 4–9) secretion induced by different IC, mAb clone refers to IC formed by VLPs and the respective mAb. Data are represented using bar graphs with dots showing the number of independent experiments, numbers above the bars are mean values, *p < 0.05, **p < 0.01, ***p < 0.001, ****p < 0.0001, one-way ANOVA followed by Tukey’s multiple comparison test with option of matched measures across one N

We further investigated whether IC properties are related to inflammasome activation. For this, we downregulated NLRP3 inflammasome activation using siRNA targeting *NLRP3* gene. First, we investigated the effect of VLPs alone on inflammasome activation. We found that targeting siRNA significantly decreased IL-1β release in VLP-treated macrophages compared to negative siRNA treatment (Fig. 6C). As expected, there were no changes in TNF-α secretion (Fig. 6D), as TNF-α secretion is dependent on NF-κB activation but not on inflammasome activation. siRNR targeting *NLRP3* did not totally downregulate NLRP3 protein expression, the decrease was approximately 50% (Fig. 6A, B). IL-1β release also decreased by approximately 50% (Fig. 6C, see mean values above the bars). We also used MCC950, which selectively inhibits NLRP3 inflammasome activation. However, it also incompletely inhibited IL-1β release (Fig. 6E). As siRNA targeting *NLRP3* treatment, MCC950 did not affect TNF-α secretion (Fig. 6F). This suggests that VLPs mediate alternative inflammatory mechanisms in addition to NLRP3 inflammasome activation. Analysing IL-1β release after treatment with IC, we found that siRNA targeting *NLRP3* significantly downregulated IL-1β secretion in all cases (Fig. 6G). These results also confirmed that IC formed by the highest affinity mAb 12F8 enhanced NLRP3 inflammasome activation (Fig. 6G).

We further analysed whether VLP-induced chemokine release depends on inflammasome activation. After downregulation of *NLRP3* expression, we found a significant reduction in CXCL2, CXCL9, CCL8 secretion (Fig. 6I–K) but not in CXCL1 (Fig. 6H). The decrease was partial, suggesting that VLP-induced secretion of inflammatory chemokines is dependent on NLRP3 inflammasome activation, but other macrophage signaling pathways are involved.

## Discussion

MAbs are prominent therapeutic tools for viral infections and other diseases as they are highly specific, can modulate the immune response and neutralise viruses [35]. The properties of therapeutic mAbs can influence their functions, for example, high-affinity mAbs ensure their low usage. Additionally, mAb subclass influences its binding to FcR and subsequent cell activation. For example, virus-specific IgG that strongly binds to activating FcγR showed high potency for the treatment of SARS-CoV-2 infection [36]. In viral infections, Abs perform important effector functions, such as Ab-dependent cellular cytotoxicity or phagocytosis of opsonised antigens [37]. Moreover, Ab-mediated Fc effector function is particularly important in protection from viral infection by modulating humoral and cellular immune responses [38]. However, Ab-dependent enhancement can occur due to IC-induced activation of the inflammatory response in FcγR-positive immune cells [39–41]. As the properties of mAbs and the antiviral activity or disease-modulating effects of formed IC are under investigation, we aimed to study macrophage response to IC composed of viral antigens and mAbs of different IgG subclasses.

We selected WUPyV-derived VLPs as they mimic native viruses in their shape and structure. In general, WUPyV is detected in patients with respiratory infections and is found in various parts of the respiratory tract, suggesting that this virus is a cause of respiratory diseases [42]. However, WUPyV pathogenicity is still debated, especially its association with severe respiratory infections [43]. Epidemiological studies showed the presence of WUPyV as a coinfection agent with other respiratory viruses, however, due to the small numbers of patients, the relation of coinfection with disease severity is inconclusive [44]. Recently, SARS-CoV-2 coinfection with WUPyV was evinced, however, additional studies are needed to show the clinical outcome of these coinfections [45]. Moreover, WUPyV capability to replicate in human airway epithelial cells [46] supports the possible contribution of this virus to severe respiratory infections in mono- or coinfection cases. As human PyVs are highly prevalent [47], their occurrence in various respiratory infections is extremely plausible. Thus, WUPyV, as a potential respiratory pathogen, is a favorable target for investigation. To uncover the possible mechanisms of PyV-related infections and coinfections, further studies on the pathogenicity of PyVs and their interaction with immune cells are needed.

Combining advanced label-free, time-resolved, sensitive techniques and immunoassays, we revealed that mAb affinity and IC characteristics influence the response of innate immune cells. Precisely determined thermodynamic parameters allowed us to conclude that the affinity of IC-forming mAb predestines NLRP3 inflammasome signaling, as only the IC formed by the highest affinity mAb (12F8) enhanced NLRP3 inflammasome activation mediated by VLPs. This mAb was of IgG2a subclass, supposed to have a very high affinity for activating FcγR [48]. Having several mouse mAbs of IgG2a subclass with different affinities, we revealed that IC-induced cell activation depends on mAb affinity and IC structure, as mAb 12F8 formed IC with higher VLP surface coverage by mAbs than other mAbs. A previously published in silico study of SARS-CoV-2 RBD-specific mAbs demonstrated that mAb affinity and ability to bind specific regions influence their functional properties, as the size and structure of epitopes determine IC stability and binding to FcR [49]. We also found that the secretion levels of inflammatory chemokines by IC-treated cells were determined by mAb affinity. Comparing mAbs of IgG2a subclass, we detected enhanced secretion of the viral infection-associated chemokine CXCL9 by IC composed of the lowest affinity IgG2a mAb. All mAbs, except those of IgG1, downregulated the release of the inflammasome activation-related chemokine CXCL2. Interestingly, the release of CCL8, an important factor of many infections for the recruitment of leukocytes [50], was upregulated only by the mAb of IgG1 subclass. This mAb (11D2) also showed the lowest influence on the secretion of other inflammatory molecules. The exceptional property of this mAb is recognition of the conformational epitope of VLPs. A particularly sensitive technique, QCM-D, allowed us to demonstrate that the formation of IC by mAb 11D2 was a highly dynamic process with conformational changes that could occur in both VLPs and mAbs. Conformational changes during antigen and Ab contact are important for IC formation, especially if they occur in CDRs [51]. Changes in Fc were also found to be critical, as structural deformations that occur during the antigen-Ab interaction can also fate Fc affinity to FcR [52]. Overall, Fc-mediated effector function is a prominent antiviral mechanism. In line with previous observations, our data show that IC-induced cellular activation varies depending on the properties of IC-forming mAbs. In addition, the advantage of our study is the use of in vivo raised mAbs that were generated by hybridoma technology after mouse immunisation with VLPs. Repeated immunisation procedures resulted in germinal center-derived B cells producing highly specific and optimised antibodies. Consequently, the generated hybridoma cells secrete highly specific mAbs that passed in vivo affinity maturation and Fc modifications and preserve the nature of the humoral immune response [53, 54]. Therefore, our results on IC-induced inflammation mimic in vivo antiviral response of innate immune cells.

Different mAbs elicit different cellular responses due to their characteristics [55]. Multiple factors affect IC-mediated inflammatory response, including density of Abs on the antigen and Ab affinity. Receptor clustering in lipid rafts of the effector cell is essential for signal transduction through FcγRs, thus, multivalent IC are the functionally appropriate ligands for the receptors. A direct contact between FcγRs and IC is increased by a greater Ab density, which promotes early signaling steps. At a fixed Ab concentration, Ab affinity toward the Ag determines how many Fc chains are displayed on the IC and are available to interact with FcγRs. Therefore, the primary signals mediated upon IC interaction with FcγRs determine cell activation leading to inflammatory molecule secretion [56]. IC of different properties may have different effect on FcγRs clustering in lipid rafts due to IC spatial organization. Thus, the size of IC defines its binding to FcγR, signal transduction and cell activation profile [55, 57]. In line with previous observations, we determined an impact of IC size on cell activation when comparing IC formed by mAbs 12F8 and 4E12 of IgG2a subclass. Only 12F8 mAb of the highest affinity enhanced VLP-induced activation of NLRP3 inflammasome. Upon IC binding, the ITAM motifs within the cytoplasmic tails of the FcγRs become phosphorylated [58]. This leads to the recruitment and activation of Syk kinase which in turn initiates downstream activation of multiple signaling kinases and production of second messengers such as inositol trisphosphate and diacylglycerol that induce calcium mobilization and protein kinase C activation. Elevated levels of intracellular calcium can directly promote the assembly of NLRP3 inflammasome [59]. Moreover, Syk activation can lead to the generation of reactive oxygen species (ROS) through the activation of NADPH oxidase [60]. ROS production has been shown to be involved in NLRP3 inflammasome activation, either directly or by inducing mitochondrial dysfunction. In addition, it was shown that active Syk deactivates downstream AMPK, which mediates excessive mitochondrial fission and leads to activation of the NLRP3 inflammasome [61]. Activation of Syk coupled to C-type lectin receptors can induce mitochondrial ROS and further activate NLRP3 inflammasome [62]. IC formed by SARS-CoV-2 S protein and specific Ab were shown to promote inflammatory cytokines and chemokines via Syk signaling pathway in macrophages [63]. This signaling pathway can be regulated by many factors, therefore, IC formed by different Abs have various effects on inflammatory responses in effector cells.

Models of IC formed by mAbs and viral antigens are important for the investigation of viral infection-related inflammation. For example, IC of the hepatitis B virus capsid and specific Abs are supposed to be generated during infection and their induced inflammation may contribute to disease progression, however, the role of IC in this long-lasting process is not fully understood [64]. In addition, liver inflammation correlates with serum IC levels, addressing the contribution of IC to immunopathology. Another interesting fact, SARS-CoV-2-infected children, having high serum levels of circulating S protein and inflammatory molecules, develop multisystem inflammatory syndrome [65]. S protein can form IC and exacerbate inflammation. Moreover, high levels of SARS-CoV-2-specific IgG were detected in severe COVID-19, and circulating IC together with excessive activation of FcγR-positive cells were found in these patients, implying that IC contributes to immunopathology [66]. This could explain the mechanisms of cytokine storm concomitant with COVID-19 [67]. We showed that IC can enhance VLP-mediated inflammatory response in macrophages. Contrary to our data, downregulation of inflammasome activation by classical IC of erythrocytes and ovalbumin antigens was demonstrated in macrophages [20]. However, the authors activated macrophages with classical inflammasome activators – silica and ATP. Nevertheless, they also used the pathogen *C. albicans*, which activates NLRP3 inflammasome, however, their IC also reduced inflammasome activation. Inflammasome activators of another origin than viral antigens and their IC may have a different effect. Evidence from previous in vitro and in vivo studies of viral infections supports our data on Ab-mediated enhancement of inflammatory response related to inflammasome activation. Furthermore, the IC effect was demonstrated with other NLRP3 inflammasome activators – α-synuclein aggregates – being important players in neuroinflammation in Parkinson’s disease [68]. IC formed by α-synuclein and Abs enhanced inflammasome activation mediated by α-synuclein in human microglia [69]. This supports our findings that IC can upregulate antigen-mediated inflammasome activation. Moreover, α-synuclein aggregates stimulate similar inflammatory cytokines (IL-1β, TNF-α, CXCL1) [70] as VLPs in our study. Another neurodegenerative disease-related oligomeric protein – amyloid beta – also induces inflammasome activation in microglia, as our previous research shows [9]. Here, we demonstrated that the phagocytosed viral antigen has the same effect as amyloid beta on microglia. Likely, IC of amyloid beta can enhance antigen-induced effects, as it was shown previously that microglia activated by IC composed of amyloid beta mediate neuronal death [71]. Thus, IC-mediated enhancement of antigen-induced signals can be provoked in macrophages by IC of various antigens.

We found that VLPs induce secretion of CXCL1 and CXCL2 chemokines that can trigger inflammasome activation in macrophages by engaging CXCR2 [72]. A previous study demonstrated that CXCL1 and CXCR2 together with NLRP3 are also upregulated in microglia under brain inflammatory conditions and are thought to be involved in NLRP3 inflammasome activation [73]. In addition, our data show that VLP-induced secretion of these chemokines can be downregulated by specific mAbs, forming IC, suggesting a potential way to diminish inflammation. CXCL1 and CXCL2 can be induced by pathogens or cytokines, including IL-1β [74]. Interestingly, comparing cells treated with different IC, which either enhanced or did not VLP-induced IL-1β release, we did not find any differences in CXCL1 and CXCL2 secretion levels. This suggests that VLP-induced levels of CXCL1 and CXCL2 were independent on IL-1β release. Nevertheless, we observed differences in IC-modulated CXCL1 and CXCL2 secretion. Only IC formed by mAbs of IgG1 subclass, which had the lowest impact on IL-1β secretion, downregulated a single chemokine (CXCL1), while other IC downregulated both chemokines. In another study, pneumococcal infection was shown to trigger the release of IL-1β following the induction of CXCL1 and CXCL2 in epithelial cell [75]. These chemokines are important for neutrophil recruitment during bacterial infection and may play a different role in viral infection. In another study, the blockade of IL-1β receptor IL-1R1 in human epithelial cells infected with rhinoviruses prevented the production of pro-inflammatory molecules, including CXCL2, demonstrating that IL-1β can be a direct inducer of CXCL2 [76]. However, we did not observe changes in CXCL2 secretion related to IC-induced unequal levels of IL-1β release, suggesting cellular response as a complex and time-dependent process.

High expression of pro-inflammatory molecules of IFN- and TNF-dependent pathways are detected during viral infections, for example, SARS-CoV-2 [77], hepatitis B [78], and HIV-1 [79]. High levels of these molecules, detected after macrophage treatment with VLPs (TNFα, IL-1β, CCL8, CXCL8, CXCL9, CXCL16, CXCL1, and CXCL2), were shown to correlate with severe COVID-19 in other studies [80–82]. Their secretion is stimulated by viral antigens in innate immune cells to recruit various immune cells to the site of infection. In viral infections, CXCL9 together with CXCL10 play a fundamental role by attracting activated T cells via CXCR3 [83]. Macrophage-derived CXCL9 and CXCL10 are important chemokines in stimulating adaptive immunity [84]. Elevated expression of the latter chemokines is also observed in PyV infection, and they are found in kidney transplant rejection, which correlates with a high prevalence of BKPyV infection in these patients [85] and indicates an association of BKPyV-induced inflammation with renal pathology. Other CXC chemokines, including CXCL1, CXCL9, and CXCL16, were also demonstrated to participate in the pathogenesis of PyV infections [86]. We revealed that our PyV-derived VLPs, mimicking intact viruses, directly stimulate the release of these chemokines in macrophages, while VLP-specific mAbs modulate their secretion.

**Fig. 7:**
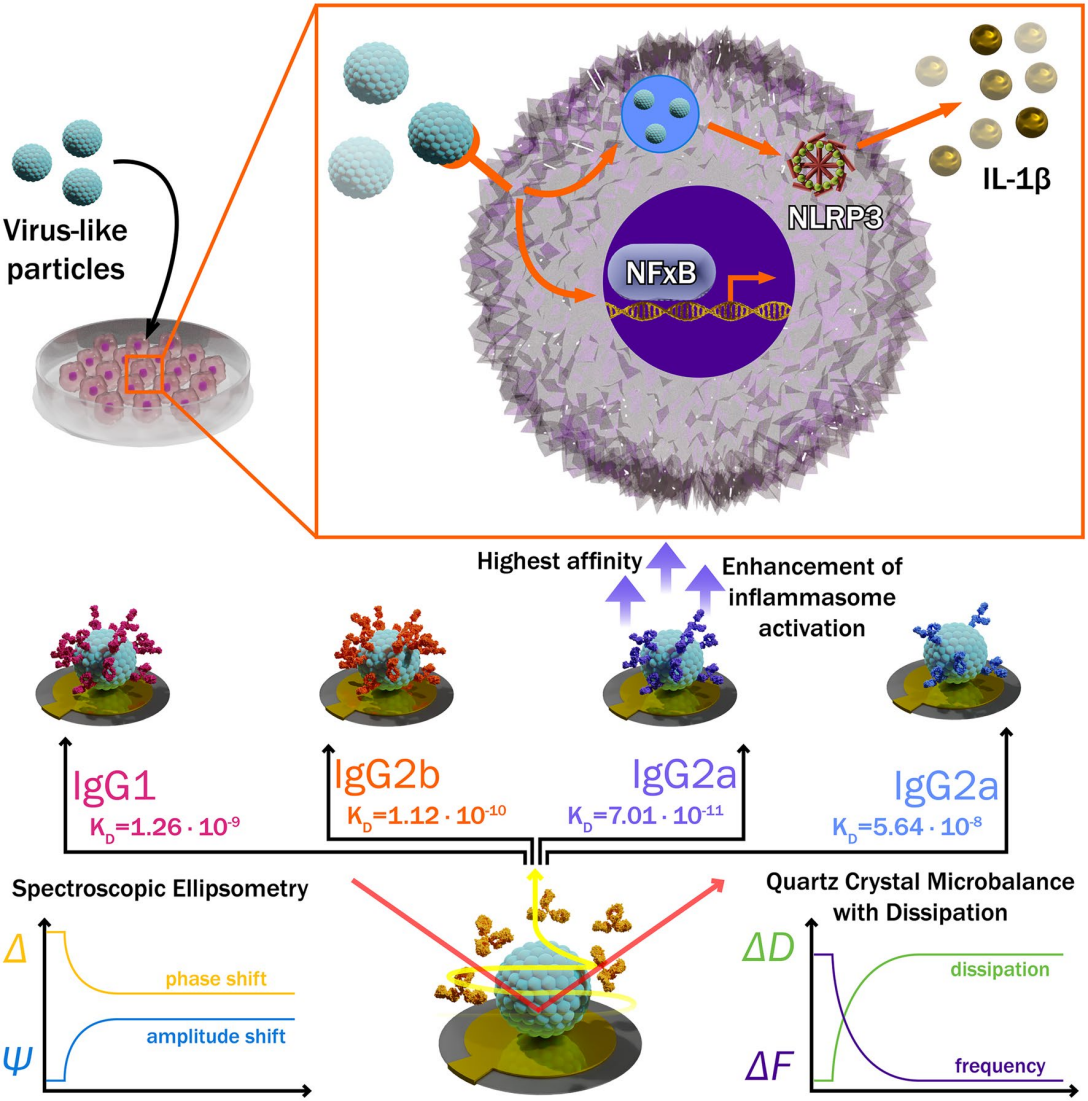
Graphical summary. Antibodies can enhance viral antigen-induced inflammasome activation in macrophages. NLRP3 inflammasome activation by IC depends on Ab affinity and the amount of Ab covering the surface of antigen. SE and QCM-D were used to characterise Ab affinity and real-time formation of IC

In this study we demonstrated that the inflammatory mechanisms of IC-mediated activation of innate immune cells depend on mAb affinity, subclass, epitope, and the properties of formed IC (Fig. 7). The identified mAb capability to enhance inflammasome activation induced by VLPs represents a potential mechanism mimicking hyperinflammation during viral infection. Previous studies and our data evince that antibody-mediated effector functions have a high impact on the outcome of viral infection. In addition, we address the need for a broader investigation of mechanisms related to antibody-mediated enhancement of the immune response, FcγR-mediated cell activation, the role of a particular IgG subclass and Ab affinity in activation signal transduction via FcγR for the development of safe and efficient vaccines as well as antibody-based therapeutics. Our findings will deepen the understanding of the antiviral immune response and could be relevant for future studies investigating IC-mediated inflammation as a consequence of viral infections or vaccine adverse effects.

### Supplementary Information

**Additional file 1: Fig. S1.** IC formation and uptake by macrophages. Cells were treated with WuPyV VLPs (20 µg/ml) and mAbs (7.5 µg/ml) for 24 h and stained with VLP-specific MAbs, cell stain (gray) and nuclear stain Hoechst33342 (blue). For IC detection, antigen and IC were stained separately. Firstly, IC were stained with secondary Ab-AlexaFluor 488 (antibody of IC, green), then anti-VLP mAb followed by secondary Ab-AlexaFluor 594 (red). Z-projects of 28 frames of composite images are shown. Z-projects were made with ImageJ using projection type standard deviation. (A) shows composite images of Z-projects and (B, C) – images of separate channels. (D, E) Images of separate channels of z = 4 (frame) of 28 frames, yellow lines show selected regions for orthogonal views represented in Fig. 4. Z-projects of composite and separate channels images are represented. 3D images were taken using ×63-oil objective with Leica TCS SP8 confocal microscope. Images have z-size 8.06 μm and 28 frames with z-step size 0.3 μm (system optimized). The scale bars – 30 μm. 11D2, 12F8, 4E12, 5H10 – mAb clones. **Fig. S2.** VLPs and IC induced different release of inflammatory molecules in macrophages. Cells were treated with VLPs (20 µg/ml) and mAbs (7.5 µg/ml) for 24 h. (A) TNF-α, (B) IL-1β and (C) IL-12/23 secretion determined by ELISA. (D) CXCL1, (E) CXCL2, (F) CXCL9, (G) CCL8, (H) CXCL16 secretion determined by ELISA. The figures represent data from Fig. 5 together with mAb alone controls. Data are represented using bar graphs or box plots with dots showing the number of independent experiments (N), *p < 0.05, **p < 0.01, ***p < 0.001, ****p < 0.0001, one-way ANOVA followed by Tukey’s multiple comparison test, for chemokines test option of matched measures across one N was used, for IL-12/23 data two-tailed unpaired t-test was used.

## Data Availability

All data used to support the findings of this study are included within the article.

## Abbreviations

Ab: Antibody
FcR: Fc receptor
IC: Immune complexes
mAb: Monoclonal antibody
N: Nucleocapsid
PyV: Polyomavirus
SE: Spectroscopic ellipsometry
QCM-D: Quartz crystal microbalance with dissipation
