# Supplementary material for: Structural properties of immune complexes formed by viral antigens and specific antibodies shape the inflammatory response of macrophages
Source: Cell Biosci. 2024 Apr 25;14:53. doi: 10.1186/s13578-024-01237-1 (PMC11046781; doi:10.1186/s13578-024-01237-1)
Supplement: Supplementary file 1 — Additional file 1: Fig. S1. IC formation and uptake by macrophages. Cells were treated with WuPyV VLPs (20 µg/ml) and mAbs (7.5 µg/ml) for 24 h and stained with VLP-specific MAbs, cell stain (gray) and nuclear stain Hoechst33342 (blue). For IC detection, antigen and IC were stained separately. Firstly, IC were stained with secondary Ab-AlexaFluor 488 (antibody of IC, green), then anti-VLP mAb followed by secondary Ab-AlexaFluor 594 (red). Z-projects of 28 frames of composite images are shown. Z-projects were made with ImageJ using projection type standard deviation. (A) shows composite images of Z-projects and (B, C) – images of separate channels. (D, E) Images of separate channels of z = 4 (frame) of 28 frames, yellow lines show selected regions for orthogonal views represented in Fig. 4. Z-projects of composite and separate channels images are represented. 3D images were taken using ×63-oil objective with Leica TCS SP8 confocal microscope. Images have z-size 8.06 μm and 28 frames with z-step size 0.3 μm (system optimized). The scale bars – 30 μm. 11D2, 12F8, 4E12, 5H10 – mAb clones. Fig. S2. VLPs and IC induced different release of inflammatory molecules in macrophages. Cells were treated with VLPs (20 µg/ml) and mAbs (7.5 µg/ml) for 24 h. (A) TNF-α, (B) IL-1β and (C) IL-12/23 secretion determined by ELISA. (D) CXCL1, (E) CXCL2, (F) CXCL9, (G) CCL8, (H) CXCL16 secretion determined by ELISA. The figures represent data from Fig. 5 together with mAb alone controls. Data are represented using bar graphs or box plots with dots showing the number of independent experiments (N), *p < 0.05, **p < 0.01, ***p < 0.001, ****p < 0.0001, one-way ANOVA followed by Tukey’s multiple comparison test, for chemokines test option of matched measures across one N was used, for IL-12/23 data two-tailed unpaired t-test was used. [file 13578_2024_1237_MOESM1_ESM.pdf]

Structural Properties of Immune Complexes Formed by Viral Antigens and Specific Antibodies Shape the Inflammatory Response of Macrophages

The running title: **The Features of Immune Complexes Fate Inflammatory Response**

Asta Lučiūnaitė[1*], Kristina Mašalaitė[1*], Ieva Plikusiene[3,4], Vincentas Maciulis[3], Silvija Juciute[2], Milda Norkienė[1] and Aurelija Žvirblienė[1]

[1]Institute of Biotechnology, Life Sciences Center, Vilnius University, Vilnius, Lithuania.

[2]NanoTechnas - Center of Nanotechnology and Materials Science, Faculty of Chemistry and Geosciences, Vilnius University, Vilnius, Lithuania.

[3]State Research Institute Center for Physical Sciences and Technology, Vilnius, Lithuania.

[4]Pharmacy and Pharmacology Center, Faculty of Medicine, Vilnius University.

[*]'These two authors contributed equally to this work.'

Corresponding author: Asta Lučiūnaitė; address: Sauletekio ave. 7, 10257 Vilnius, Lithuania; tel. no.: (+370 5) 223 43 74; e-mail: asta.luciunaite@bti.vu.lt

Supplementary Information

Supplemetary Figures

Supplemetary Figure Legends

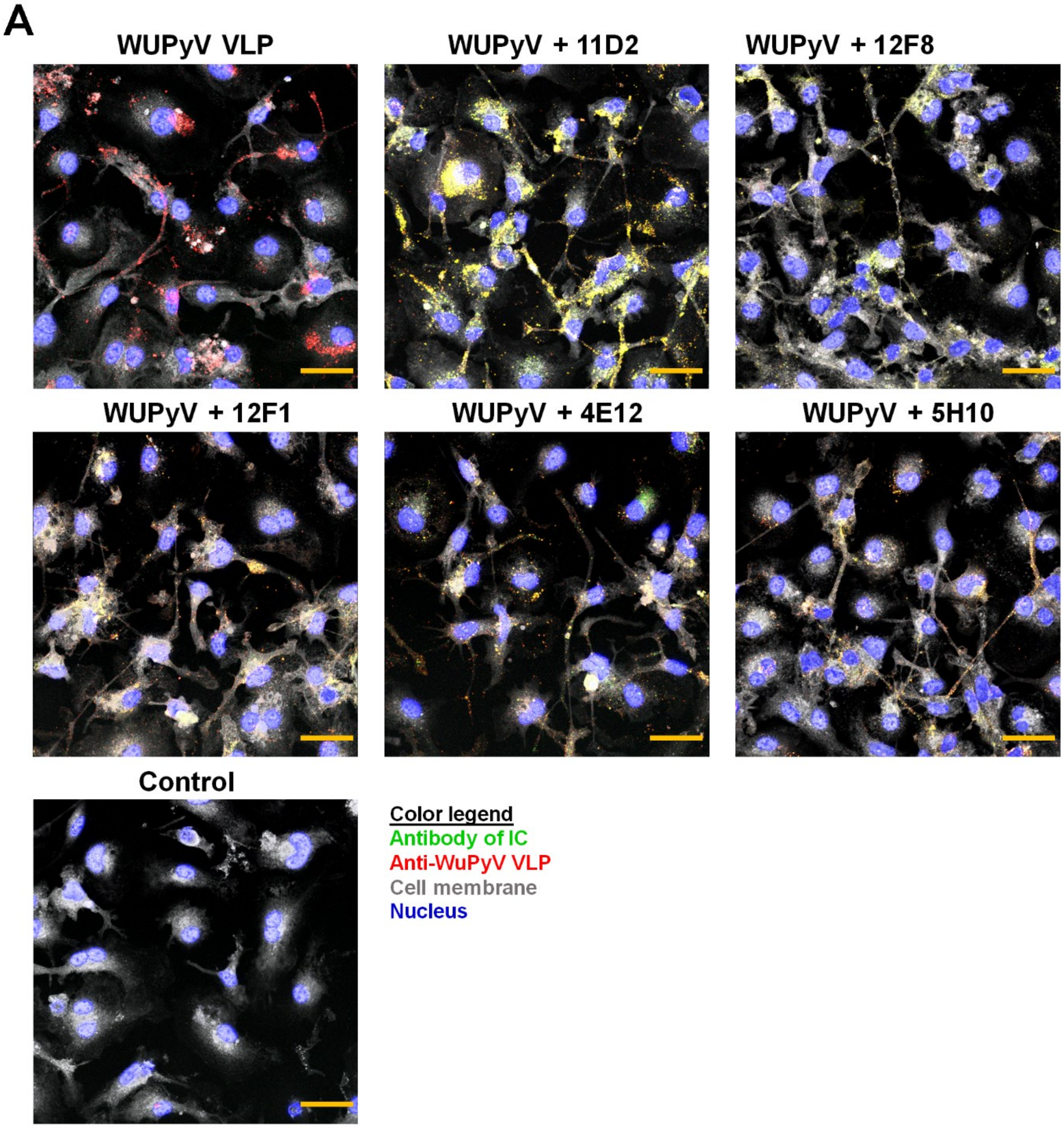
A
WUPyV VLP
WUPyV + 11D2
WUPyV + 12F8
WUPyV + 12F1
WUPyV + 4E12
WUPyV + 5H10
Control
Color legend
Antibody of IC
Anti-WuPyV VLP
Cell membrane
Nucleus

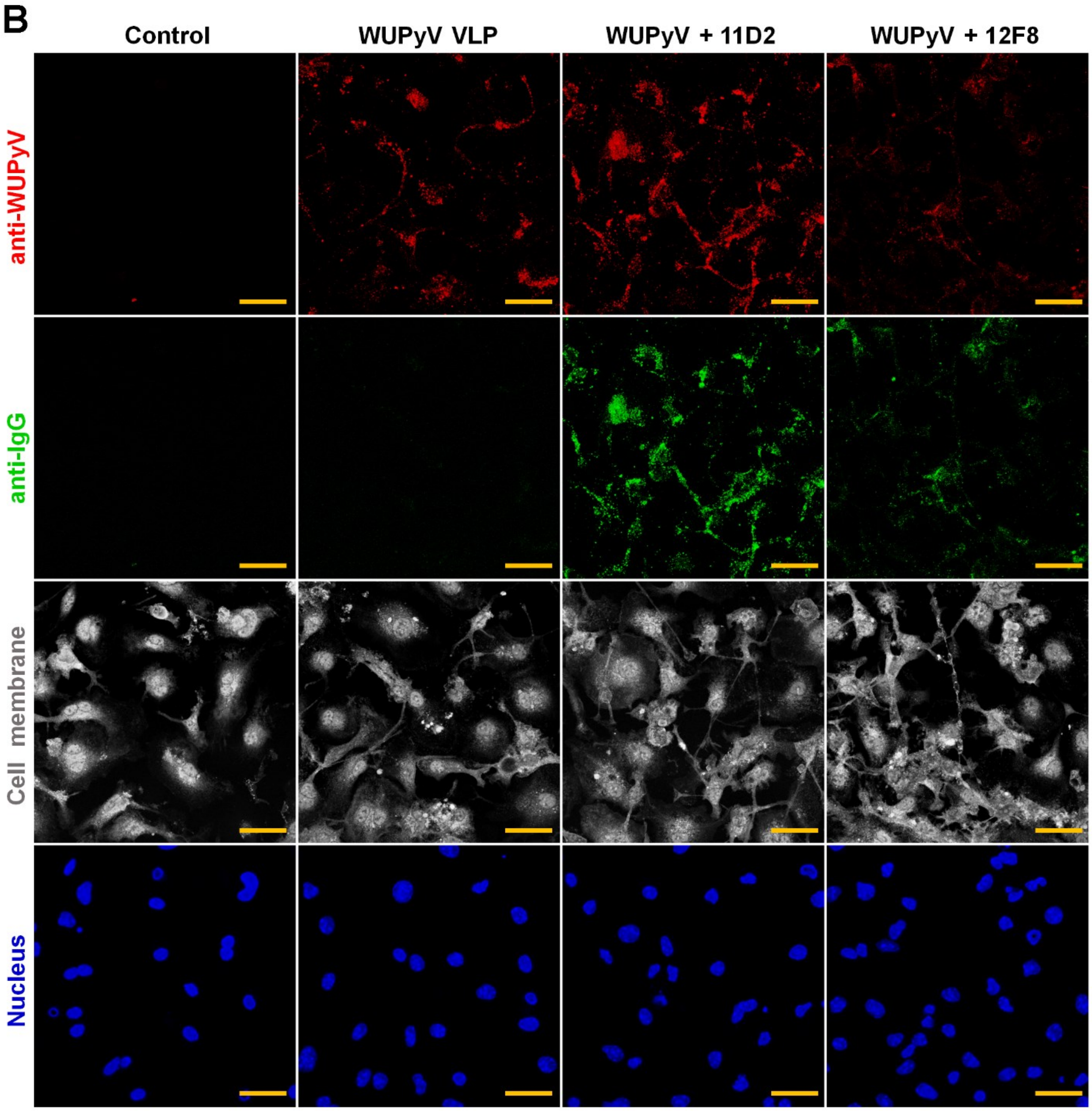
B
Control
WUPyV VLP
WUPyV + 11D2
WUPyV + 12F8
anti-WUPyV
anti-IgG
Cell membrane
Nucleus

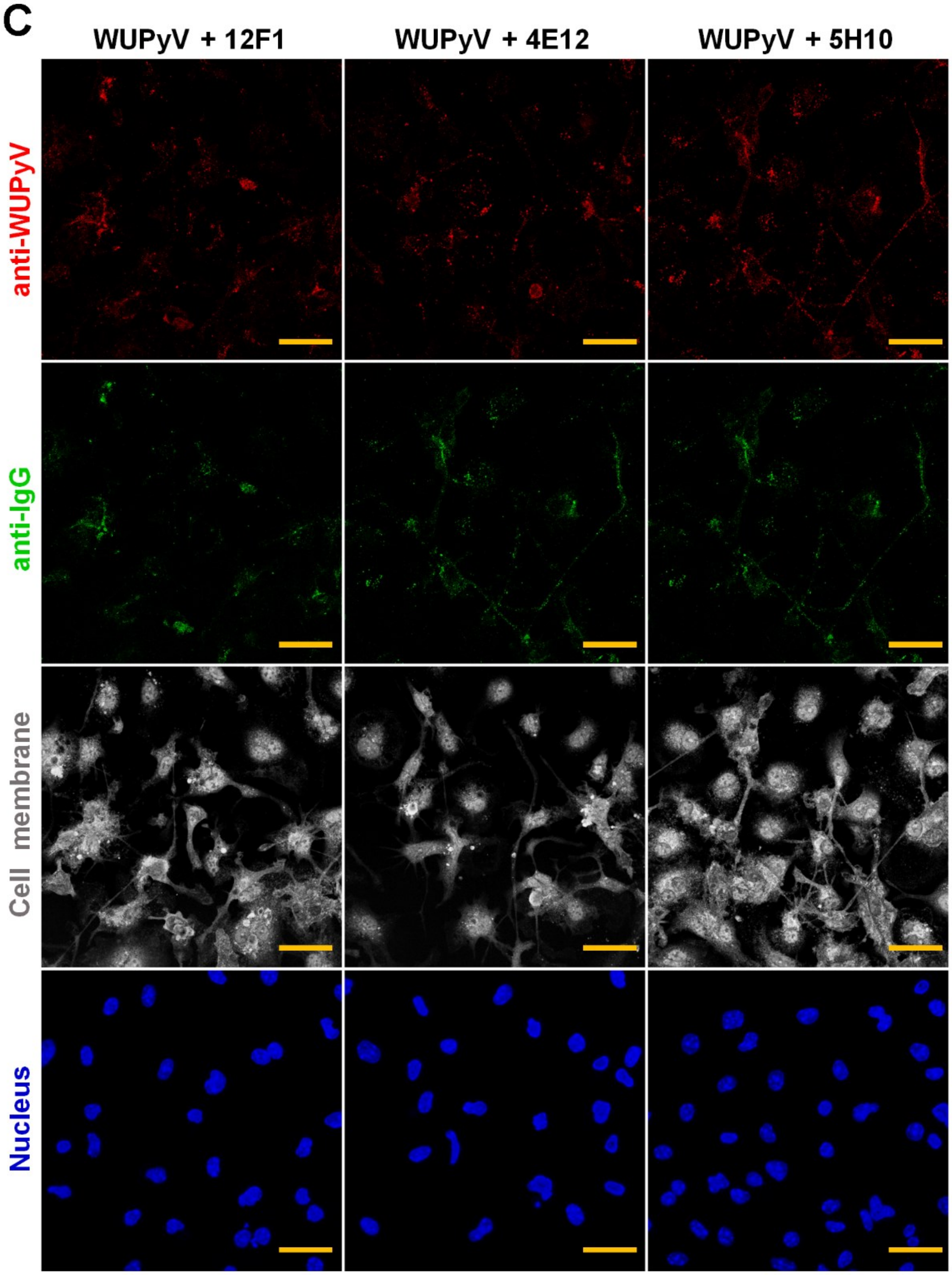
C
WUPyV + 12F1
WUPyV + 4E12
WUPyV + 5H10
anti-WUPyV
anti-IgG
Cell membrane
Nucleus

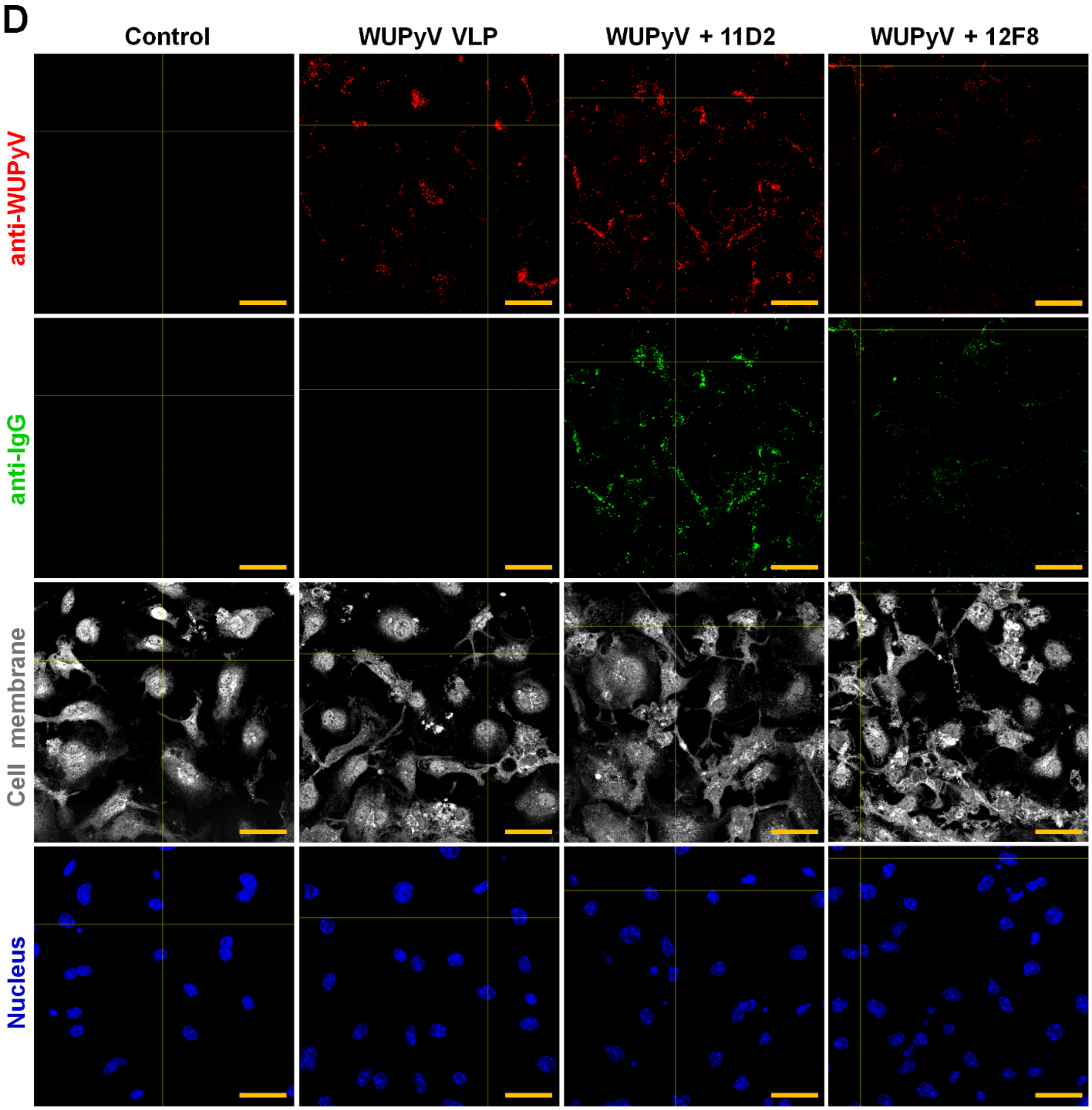
D
Control
WUPyV VLP
WUPyV + 11D2
WUPyV + 12F8
anti-WUPyV
anti-IgG
Cell membrane
Nucleus

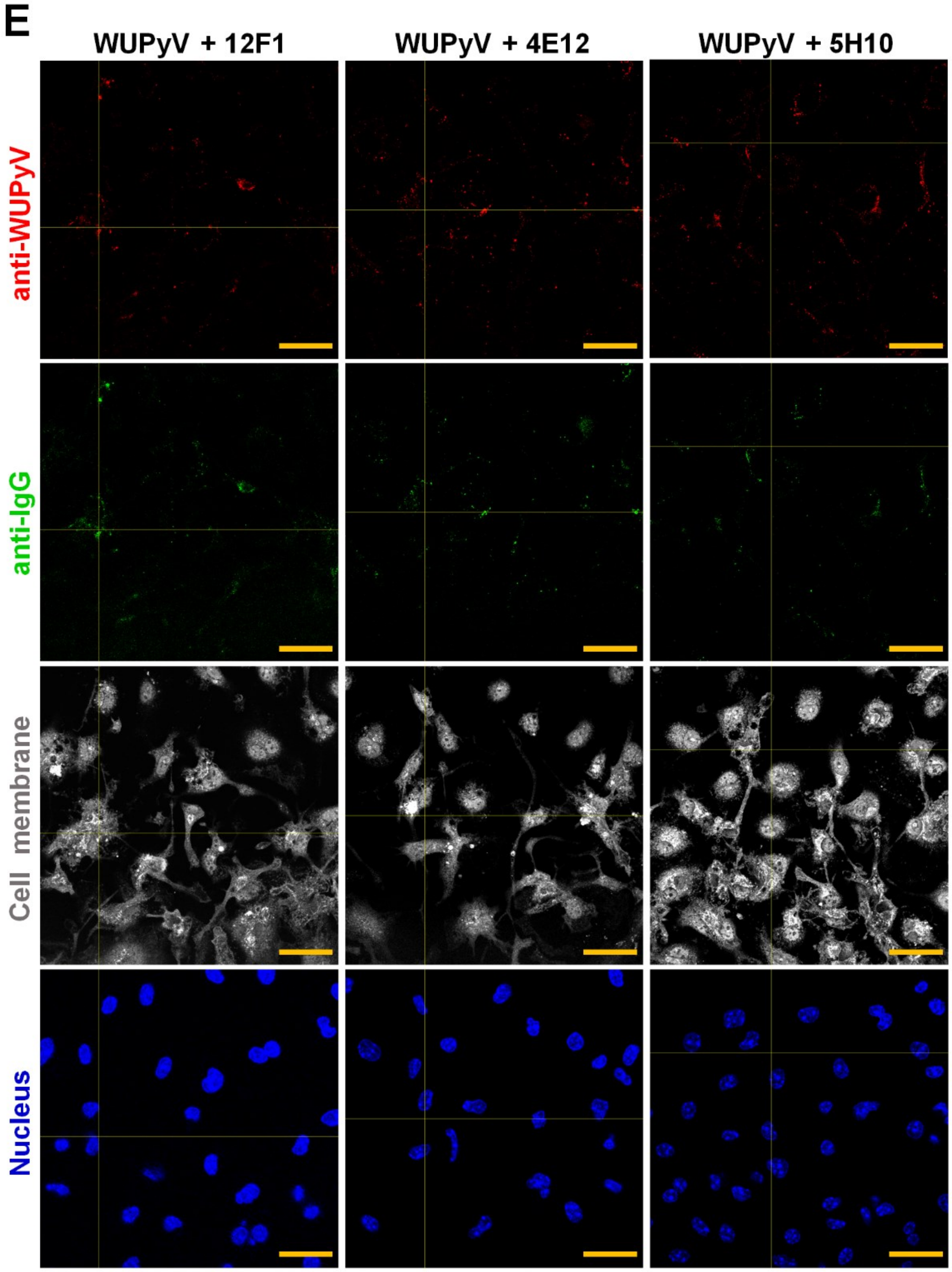

Supplementary Fig. S1. IC formation and uptake by macrophages. Cells were treated with WuPyV VLPs (20 µg/ml) and mAbs (7.5 µg/ml) for 24 h and stained with VLP-specific MAbs, cell stain (gray) and nuclear stain Hoechst33342 (blue). For IC detection, VLPs and IC were stained separately. Firstly, IC were stained with secondary Ab-AlexaFluor 488 (antibody of IC, green), then anti-VLP mAb followed by secondary Ab-AlexaFluor 594 (red). Z-projects of 28 frames of composite images are shown. Z-projects were made with ImageJ using projection type standard deviation. (A) shows composite images of Z-projects and (B, C) – images of separate channels. (D, E) Images of separate channels of z = 4 (frame) of 28 frames, yellow lines show selected regions for orthogonal views represented in Fig. 4. Z-projects of composite and separate channels images are represented. 3D images were taken using ×63-oil

objective with Leica TCS SP8 confocal microscope. Images have z-size 8.06 µm and 28 frames with z-step size 0.3 µm (system optimized). The scale bars – 30 µm. 11D2, 12F8, 4E12, 5H10 – mAb clones.

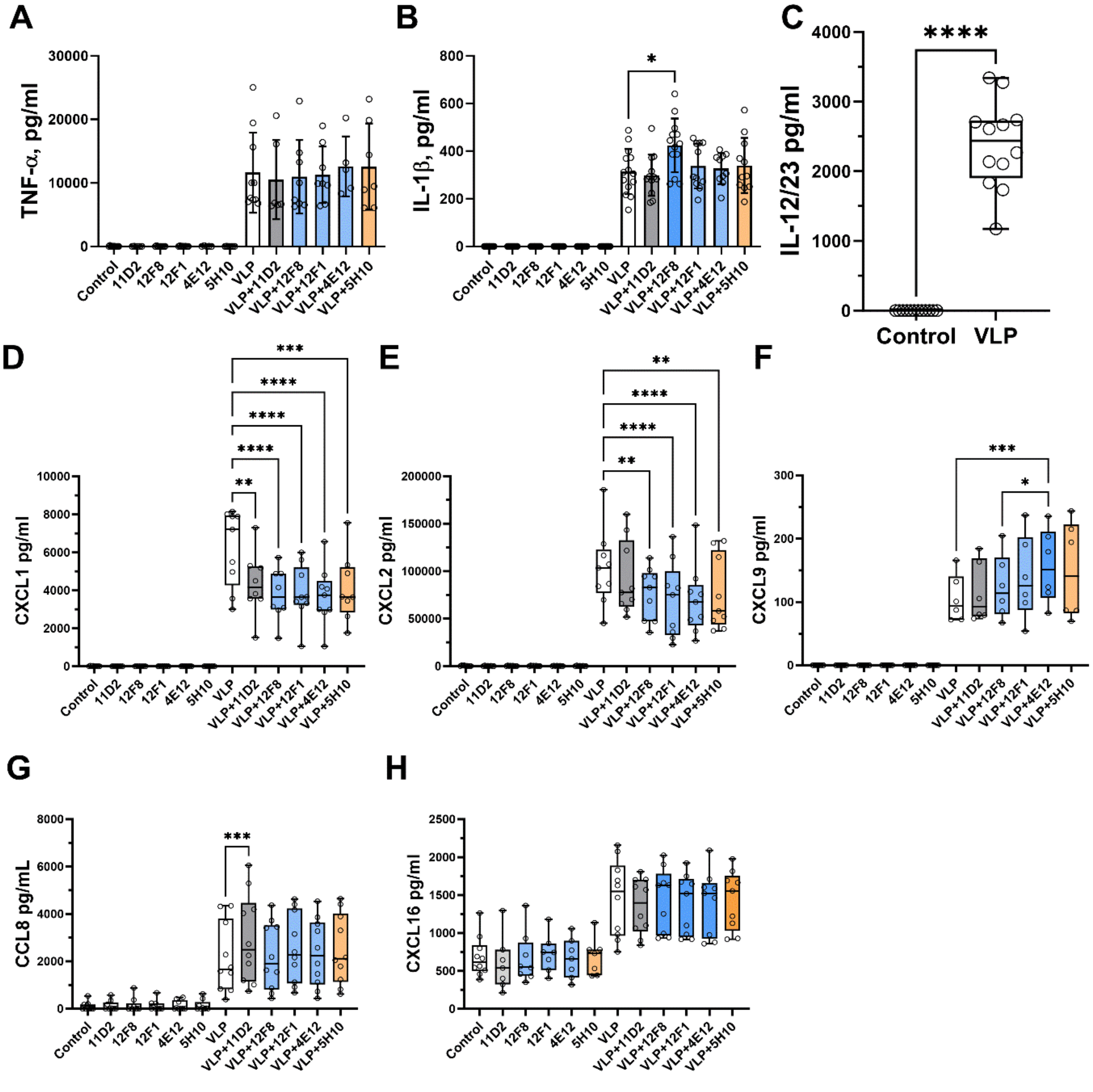

Supplementary Fig. S2. VLPs and IC induced different release of inflammatory molecules in macrophages. Cells were treated with VLPs (20 µg/ml) and mAbs (7.5 µg/ml) for 24 h. (A) TNF-α, (B) IL-1β and (C) IL-12/23 secretion determined by ELISA. The figures represent data from Fig. 5 together with mAb alone controls. (D) CXCL1, (E) CXCL2, (F) CXCL9, (G) CCL8, (H) CXCL16 secretion determined by ELISA. Data are represented using bar graphs or box plots with dots showing the number of independent experiments (N), *$p < 0.05$, **$p < 0.01$, ***$p < 0.001$, ****$p < 0.0001$, one-way ANOVA followed by Tukey's multiple comparison test, for chemokines test option of matched measures across one N was used, for IL-12/23 data two-tailed unpaired t-test was used.
